# Defect Structure and Anion Conduction in Lanthanum Oxychloride Solid Solutions Revealed by X‑ray Excited Optical Luminescence and Auger Emission

**DOI:** 10.1021/acs.chemmater.5c01868

**Published:** 2025-12-03

**Authors:** Jingxiang Cheng, Victor Alexander Gomez, Jaime R. Ayala, Alice R. Giem, Arnab Maji, Shruti Hariyani, Lucia Zuin, Sarbajit Banerjee

**Affiliations:** † Department of Chemistry, 14736 Texas A&M University, College Station, Texas 77843-3012, United States; ‡ Department of Material Science and Engineering, 14736 Texas A&M University, College Station, Texas 77843-3012, United States; § Laboratory for Inorganic Chemistry, Department of Chemistry and Applied Biosciences, 27219 ETH Zurich, Vladimir-Prelog-Weg 2, CH-8093 Zürich, Switzerland; ∥ Laboratory for Battery Science, PSI Center for Energy and Environmental Sciences, Paul Scherrer Institute, Forschungsstrasse 111, CH-5232 Villigen PSI, Switzerland; ⊥ Canadian Light Source, University of Saskatchewan, Saskatoon, SK S7N 2 V3, Canada

## Abstract

Ion transport in crystalline solids is governed by the interplay between mobile ions and the host framework; ion mobility is facilitated by local structural distortions, dynamical evolution in the local coordination environment of the mobile ion, and coupling with lattice phonons. Ion dynamics are strongly influenced by the size and hardness of the mobile ion and the rigidity and polarizability of the host framework, as well as by the nature and concentration of intrinsic or engineered defects. While the design of superionic cation conductors has attracted much recent attention, mechanisms of bulk anion diffusion and their coupling with defect dynamics remain incompletely understood. In this work, we examine the role of anion vacancies introduced by site-selective aliovalent modification of LaOCl in modifying local structure and modulating halide-ion diffusion. Distinctively, we use Dy- and Tb-ion chromophores situated on the La sublattice of LaOCl as local structure probes of the modulation of local symmetry, defect-mediated trap states, and phonon relaxation pathways using X-ray excited optical luminescence (XEOL) and Auger emission. Upon excitation of La core-levels, XEOL measurements reveal two distinct dissipative channels: (a) excitation at the giant resonance engenders nonradiative emission of Auger electrons, which is intensified with increasing concentration of chloride vacancies; (b) excitation above or below the giant resonance sensitizes La → Dy/Tb energy transfer, followed by activation of radiative recombination channels at the luminescent chromophores. Stronger blue emission from defect-mediated midgap states and thermally populated states of Dy and Tb dopants is observed. As such, the ratio of luminescence between thermally populated and thermalized states and the excitation range of Auger emission provides a sensitive measure of the concentration of chloride vacancies and maps with high fidelity to anion conductivity. Ionic conductivity in the range of 2.76 × 10^–5^–4.3 × 10^–5^ S/cm can be achieved at 300 °C at Cl vacancy concentrations of ca. 20 at. %, which holds promise for utilization as thermally robust and low electronic conductivity ceramic solid electrolytes of halide-ion batteries proposed as sustainable and safe alternatives to current electrochemical energy storage technologies. In addition to establishing an XEOL analytical probe of defect dynamics, the results illuminate key mechanistic understanding and provide design principles for site-selective modification and tuning of defect concentrations to modulate phonon band structure and promote anion diffusion.

## Graphical abstract

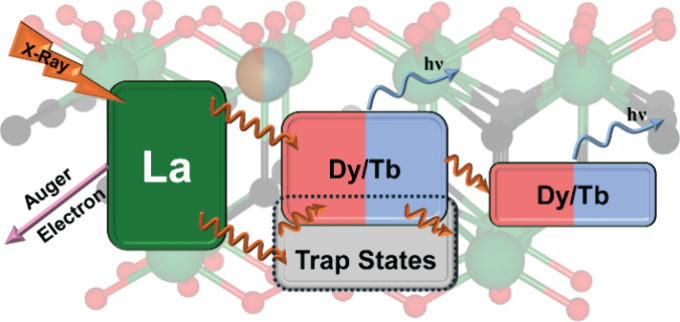

## Introduction

The migration of ions through periodic inorganic solids is mediated by a variety of mechanisms that entail local distortion of the crystal lattice, manifest continuous modification of the coordination environment of the mobile ion, and are strongly dependent on the interactions between mobile ions and the host framework.^1–3^ Ion–crystal-lattice interactions are governed by the hardness of the mobile ion, the polarizability and electronic structure of the host framework, and the structure and dynamics of intrinsic and engineered defects. Superionic conduction most commonly engages both rotational and vibrational modes, encompassing both cation and anion sublattices, and involving the correlated migration of multiple ions and point defects.^4–6^ While much recent attention has focused on the design of superionic cation conductors, mechanisms of bulk anion diffusion in solids remain incompletely understood.^7–10^ However, it is increasingly apparent that anion migration in periodic solids requires substantial deformation of the host lattice and coupling to point defects, which are modulated in large measure by the specifics of phonon dispersion in the crystal lattice, formation of collective modes in concert with point defects, and charge delocalization across the host framework.^11,12^ In this work, we examine the role of anion vacancies introduced by site-selective modification in modifying local structure and modulating halide-ion diffusion in lanthanum oxychloride. Distinctively, we use lanthanide chromophores as probes to investigate the modulation of local symmetry, phonon-mediated relaxation pathways, midgap trap states, and coordination environments as measured using X-ray excited optical luminescence and Auger emission in conjunction with Cl L-edge X-ray absorption near-edge structure (XANES) spectroscopy upon the introduction of halide vacancies. As such, we correlate defect structure and concentration to halide-ion conductivity.

Rare-earth oxyhalides (REOX, X = F, Cl, Br, and I) represent an intriguing class of halide solid electrolytes.^13–19^ These mixed anion materials manifest considerable compositional and structural diversity characterized by distinctive halide slabs that manifest record values of halide-ion conductivity, particularly for chloride, bromide, and iodide ions.^18–21^ Such solid electrolytes are of particular relevance to halide-ion batteries that represent an orthogonal construct to conventional Li-ion batteries and can potentially ameliorate the supply chain and safety challenges of the latter based on the utilization of earth-abundant halide-ions as charge carriers and by mitigating the need for metal electrodeposition.^22,23^ Since the seminal work from Imanaka in 2002, the thermal and chemical stability as well as hardness of LaOCl makes it an particularly intriguing potential solid electrolyte for high-temperature solid-state ceramic chloride batteries.^24,25^ Where many halide systems are highly corrosive and incompatible with common electrode materials and current collectors, LaOCl remains chemically stable, providing a robust platform for investigating Cl-ion transport. LaOCl has a wide bandgap of 5.54 eV with an expansive voltage stability window without decomposition or volatilization of halide species.^26^ An an exceptional dielectric breakdown field strength of >10 MV cm^–1^ has been reported for thin nanosheets with minimal electronic conductivity.^27^ Early lanthanides crystallize in a PbFCl structure with distinctive (LaO)^+^ and Cl^–^ slabs wherein the latter is conducive to ion conduction mediated by soft interlayer phonon modes.^17,19,28–31^ Aliovalent site-selective modification of LaOCl on the lanthanum sublattice such as with alkaline-earth ions introduces chloride vacancies. Such vacancies can mediate ion conduction through traditional Schottky vacancy hopping mechanisms, as well as likely through softening of collective lattice phonon modes.^13,14,18,20,21^ In contrast, homovalent substitution of La with trivalent rare-earth activators such as Dy and Tb installs sensitive probes of local structure that afford multiple radiative relaxation channels. The luminescent response of these probes at low concentrations reveals details of modification of local structure resulting from increasing concentration of anion vacancies and improved anion mobility. The matlockite crystal structure of LaOCl can accommodate a significant concentration of anion vacancies without phase transitions or degradation, enabling systematic examination of the evolution of ion conductivity with increasing dopant concentration.^13,14^ As such, while no longer the record chloride-ion solid electrolyte, LaOCl is a robust and versatile platform for systematic investigations of chloride-ion transport mechanisms and for mechanistic studies correlating defect structure with anion mobility in high-temperature chloride-ion batteries.

Herein, we report the synthesis of LaOCl alloyed with varying concentrations of Ca-ions on the cation sublattice and with Dy/Tb as optical probes. We demonstrate the use of X-ray excited optical luminescence (XEOL) spectroscopy as well as X-ray excited Auger emission as powerful means to probe halide vacancies within the LaOCl crystal lattice. 2D XANES–XEOL maps reveal detailed insights into defect types, local symmetry, and its evolution with site-selective modification.^32,33^ We map the engineered defects to ion transport and thereby establish useful spectroscopic proxies for anion conductivity. The results provide insights into anion conduction mechanisms in a leading class of Cl-ion conductors.

## Experimental Section

### Materials

La_2_O_3_ (≥99.9%), NH_4_Cl (≥99.9%), and (COO)_2_Ca·H_2_O (≥99.0%) were purchased from Millipore Sigma. Dy_2_O_3_ (≥99.9%) and Tb_2_O_3_ (≥99.9%) were purchased from ThermoFisher Scientific. All precursors were dried under a steady flow of N_2_ overnight before use.

### Synthesis

LaOCl powders were prepared using a solid-state reaction by reacting stoichiometric amounts of La_2_O_3_ and NH_4_Cl by adapting a previous method reported in the literature as per:^34–36^

$$La_{2}O_{3}\left(s\right)+2NH_{4}\mathrm{Cl}\left(s\right)\to 2\mathrm{LaOCl}\left(s\right)+2NH_{3}\left(g\right)+H_{2}O\left(g\right)$$ 1

Site-selective modification was achieved by supplanting La_2_O_3_ with various molar ratios of the precursors to be substituted in the cation sublattice. Specifically, (COO)_2_Ca·H_2_O was used for aliovalent substitution. To prepare Ca_*x*_La_1–*x*_OCl_1–*x*_, 4.5 mmol of La_2_O_3_, 9 mmol of NH_4_Cl and 1 mmol of calcium oxalate powder were mixed thoroughly with a mortar and pestle and placed in a covered alumina crucible. The mixtures were heated at a controlled heat rate of 5 min/°C to 400 °C for 2 h to evolve NH_3_ and subsequently to 850 °C for 12 h in an MTI GSL-1700 high-pressure tube furnace under an ambient air environment. A total of four Ca-alloyed variants were prepared using (COO)_2_Ca·H_2_O/La_2_O_3_ molar ratios of 5, 10, 20, and 30% that can be expressed in the Kroger–Vink notation as (Ca_La_^′^)_*x*_(La_La_)_1–*x*_O­(Cl_Cl_)_1–*x*_(V_Cl_^·^)_*x*_ (M = Ca) as per:

$$\left(1-x\right)La_{2}O_{3}\left(s\right)+\left(2-2x\right)NH_{4}\mathrm{Cl}\left(s\right)+\left(2x\right){\left(\mathrm{COO}\right)}_{2}\mathrm{Ca}\left(s\right)+xO_{2}\left(g\right)\to 2{\mathrm{La}}_{1-x}{\mathrm{Ca}}_{x}\mathrm{OC}l_{1-x}\left(s\right)+\left(2-2x\right)NH_{3}\left(g\right)+\left(1-x\right)H_{2}O\left(g\right)+4xCO_{2}\left(g\right)$$ 2

The Dy- and Tb-alloyed samples were prepared in a similar method using a reaction temperature of 1050 °C instead of 850 °C with Dy_2_O_3_ and Tb_2_O_3_ as the precursors to compare particles with similar primary crystallite dimensions. The elevated synthesis temperature was employed to ensure homogeneous site-selective modification on the La sublattice, as lower reaction temperatures yield considerable compositional heterogeneity of Dy and Tb alloying. The undoped LaOCl sample was synthesized at 850 °C rather than 1050 °C to mitigate extensive anisotropic sintering into extended nanosheets observed in past work.^17^ Notably, Dy- and Tb-alloyed samples synthesized at 1050 °C show homogeneous alloying (vide infra) without extensive agglomeration and sintering. The selected thermal profiles ensure that observed differences in properties can be traced to Dy/Tb incorporation rather than particle size or microstructural effects as confounding factors. To prepare LaOCl doped with 1 at. % Dy on the La sublattice, 0.05 mmol of Dy_2_O_3_, 4.45 mmol of La_2_O_3_, 9 mmol of NH_4_Cl, and 1 mmol of calcium oxalate were thoroughly mixed using a mortar and pestle. The resulting mixture was transferred to a covered alumina crucible and subjected to heat treatment in an MTI GSL-1700 high-temperature tube furnace under ambient air. The sample was heated at a controlled rate of 5 °C/min to 400 °C and held for 2 h to facilitate NH_3_ release, followed by heating to 1050 °C and holding for 12 h to complete the reaction, yielding compositions that can be expressed in the Kroger-Vink notation as (Ca_La_^′^)_*x*_(M_La_)_*y*_(La_La_)_1–*x*−*y*_O­(Cl_Cl_)_1–*x*_(V_Cl_^·^)_*x*_ (M = Dy and Tb) as per:

$$\left(1-x-y\right)La_{2}O_{3}\left(s\right)+yM_{2}O_{3}\left(s\right)+\left(2-2x-2y\right)NH_{4}\mathrm{Cl}\left(s\right)+\left(2x\right){\left(\mathrm{COO}\right)}_{2}\mathrm{Ca}\left(s\right)+xO_{2}\left(g\right)\to 2{\mathrm{La}}_{1-x-y}M_{y}{\mathrm{Ca}}_{x}\mathrm{OC}l_{1-x}\left(s\right)+\left(2-2x-2y\right)NH_{3}\left(g\right)+\left(1-x-y\right)H_{2}O\left(g\right)+4xCO_{2}\left(g\right)$$ 3

### Characterization

Powder X-ray diffraction (XRD) patterns were acquired using a Bruker-AXS D8 Vario X-ray powder diffractometer with a Cu Kα radiation source (λ = 1.5418 Å) in the 2θ range from 5 to 90° at a step size of 0.003°. Rietveld refinements of powder XRD patterns were performed using GSASII.^37^

High-resolution transmission electron microscopy (HRTEM) images and selected area electron diffraction (SAED) patterns were acquired using an FEI Tecnai G2 F20 ST and FEI Titan Themis 300 instrument operated at an accelerating voltage of 300 kV. The LaOCl powder was dispersed in 2-propanol; aliquots from the dispersion were deposited onto 300-mesh carbon-coated Cu grids for imaging. Particle size distribution analyses were conducted on 160 individual nanoplates manually measured using ImageJ.

Elemental concentrations of Tb, Dy, La, Ca, and Cl were determined by comparator instrumental neutron activation analysis (INAA). Aliquots of 1–2 mg of powder samples were weighed and transferred into precleaned low density polyethylene (LDPE) irradiation vials. Multielement calibrators were prepared from high-purity La_2_O_3_ (Johnson Matthey, 99.9%) and from dried aliquots of aqueous Tb, Dy, La, Mg, Ca, Sr, and Cl solution standards (Inorganic Ventures, ISO 17025 certified). Approximately 0.7 g of high-purity graphite powder was added to each vial, and the irradiation vials were heat-sealed with a soldering iron. The contents of each vial were agitated by rolling and tilting the vials. Neutron irradiations of 30 s were performed using the Texas Engineering and Experiment Station 1 MW TRIGA reactor at a nominal thermal neutron fluence rate of 9.1 × 10^12^ cm^–2^ s^–1^. Following 270 s decay intervals, gamma-ray spectra were acquired for 500 s using an HPGe detector. The data reduction was performed using the NAA software package from Canberra Industries.

La N_2_- and Cl L_2,3_ XANES spectra were concurrently acquired at the Variable Line Spacing-Plane Grating Monochromator (VLS-PGM) beamline of the Canadian Light Source (CLS) in Saskatoon, SK with an energy resolution *E*/Δ*E* > 10,000.^38^ Powder samples were adhered to carbon tape and all spectra were recorded at room temperature, with a step size of 0.1 eV and a dwell time of 1 s in a sample chamber maintained below 1 × 10^–8^ Torr. In collecting XANES spectra, total fluorescence yield (FLY)^39^ was recorded with an microchannel plate (MCP) detector, whereas the partial fluorescence yields was recorded with the Silicon Drift Detectors (SDD) with a longer dwell time of 4 s. All spectra recorded were normalized with respect to the incident photon flux (*I*_0_), which in turn was recorded by monitoring the current emitted by a metallic Ni mesh (90% transmission) located just upstream of the sample.

XEOL spectra were acquired concurrently with acquisition of XANES spectra at the VLS-PGM beamline (*E*/Δ*E* > 10,000) of the CLS.^38^ Total fluorescence yield (FLY) and XEOL data were acquired concurrently. XANES spectra were acquired using VLS-PGM’s high-energy grating by scanning the energy range from 90–170 eV with a step size of 0.1 eV. The pressure within the sample chamber was <1 × 10^–8^ Torr. The FLY signal was recorded using a microchannel plate detector.^39^ All spectra were normalized to the intensity of the photon beam as measured by the drain current monitored at a nickel mesh (with a transmission of 90% in the relevant energy range) situated upstream of the sample. The total luminescence data were collected using an Ocean Optics QE65000 monochromator with a fiber-optic feed-through over a wavelength range of 350–750 nm. The XEOL spectra at fixed photon energies were collected with a dwell time of 5 s. For every scan, the beam was moved to a new sample spot to minimize radiation damage. Characteristic luminescence signatures of divalent Tb^2+^/Dy^2+^ species are not detected in XEOL spectra (vide infra). The undoped and alloyed powder samples were adhered to carbon tape prior to mounting within the sample chamber. All measurements were performed at room temperature.

Room temperature photoluminescence spectra were obtained employing a Horiba Fluoromax-4 fluorescence spectrophotometer with a 75 W xenon arc lamp for excitation. Samples were prepared for PL measurements by casting onto a quartz slide (Chemglass) after mixing the powders into a silicone resin (GE Silicones, RTV615).

### First-Principles Calculations

Geometry optimizations were carried out using DFT as implemented in the Vienna Ab-initio Simulation Package (VASP) for all alloyed LaOCl configurations.^40,41^ Brillouin zone integration was performed using a 6 × 6 × 6 Monkhorst–Pack mesh.^42^ The projector-augmented wave formalism was used to capture electron–ion interactions. Electron exchange and correlation were addressed using the generalized gradient approximation based on the Perdew–Burke–Ernzerhof functional (PAW-GGA-PBE).^43–45^ Electronic self-consistent loop and ionic relaxation loops were adjusted to be <10^–5^ and 10^–4^ eV, respectively.

Vacancy formation energies were determined by calculating the difference in total energy between the pristine structure and a modified structure embedding a vacancy. This vacancy was created by displacing the atom 3 Å from the topmost layer on the slab to avoid charge imbalance.^46–48^ The vacancy formation energy, *E*_v_, was calculated using the total energy per atom (*n*) from the pristine structure *E*_P_ subtracted by the total energy per atom of the defective structure (*E*_D_): *E*_V_ = (*E*_P_ – *E*_D_)/*n*.

A single-point energy calculation was performed to calculate the projected density of state (pDOS) with Local Orbital Suite Toward Electronic-Structure Reconstruction (LOBSTER).^49,50^ Bunge’s description for the local basis functions were used for the pDOS calculation with 6*s* and 5*d* orbitals for lanthanum, 2*s* and 2*p* orbitals for oxygen, and 3*s* and 3*p* orbitals for chlorine.

The climbing-image nudged elastic-band (CI-NEB) method using a 107-atom supercell (12.30 Å × 12.30 Å × 30.79 Å) was used to calculate the migration barrier along the minimum energy pathway.^51^ The same simulation parameters from geometry optimizations were applied except the Brillouin zone integration grid was set to the gamma point (1 × 1 × 1); the ionic optimization loop was used in conjunction with a force optimizer with a convergence criterion equal to 0.03 eV/Å.^52^

### Electrochemical Measurements

Electrochemical impedance spectroscopy (EIS) was carried out using Gamry ref-620 potentiostats and a BioLogic HTSH-1100 high-temperature sample holder. The sample powder was pressed into a pellet of 12 mm in diameter and ca. 1 mm in thickness with 8 tons of pressure using a MSE PRO benchtop automatic laboratory press. Next, the pellets were coated with platinum paste from Sigma-Aldrich (99.9%) and sintered at 400 °C for 8 h under N_2_ flow in Thermo Scientific Thermolyne benchtop muffle furnace. The AC conductivity (σ) of the pellet was measured in the frequency between 1 Hz to 13 MHz at temperatures between 25 and 400 °C. The data obtained were processed with EC-lab software to calculate conductivity.

## Results and Discussion

### Synthesis, Structural Characterization, and Defect Structure

LaOCl crystallizes in a tetragonal matlockite PbFCl-type structure with the space group *P*4/*nmm* (Figure 1A).^19^ Later lanthanides adopt a mixture of SmSI and YOF structure-types.^17,53,54^ In the PbFCl structure, (LaO)^+^ layers are separated by chloride layers, creating a structural framework in which lanthanum ions occupy the centers of monocapped square antiprisms. Each lanthanum ion is coordinated to four oxide ions in the lower layer and four Cl ions in the upper layer; in this configuration, the 9-fold La coordination environment is completed by an additional chloride ion from an adjacent layer. Aliovalent substitution such as with Mg, Ca, and Sr replaces the La^3+^ ions with a divalent alkaline-earth cation and introduces chloride vacancies in the anion layer (Figure 1B). Indeed, DFT calculations of vacancy formation energies reveal a strong preference for Cl vacancies (0.019 eV per atom), as compared to La (0.091 eV/atom), Ca (0.11 eV/atom), and O (0.12 eV/atom) vacancies. The preference for increasing chloride vacancies with aliovalent substitution is further corroborated by NAA analyses of cation and anion stoichiometries as provided in Table S1.^29^ Table S1 also lists the atomic concentration of chloride vacancies inferred from the NAA measurements. The apparent Cl excess observed in our measurements is reasonably attributed to the proclivity of LaOCl to preferentially expose low-energy Cl-terminated surfaces.^55^

**Figure 1:**
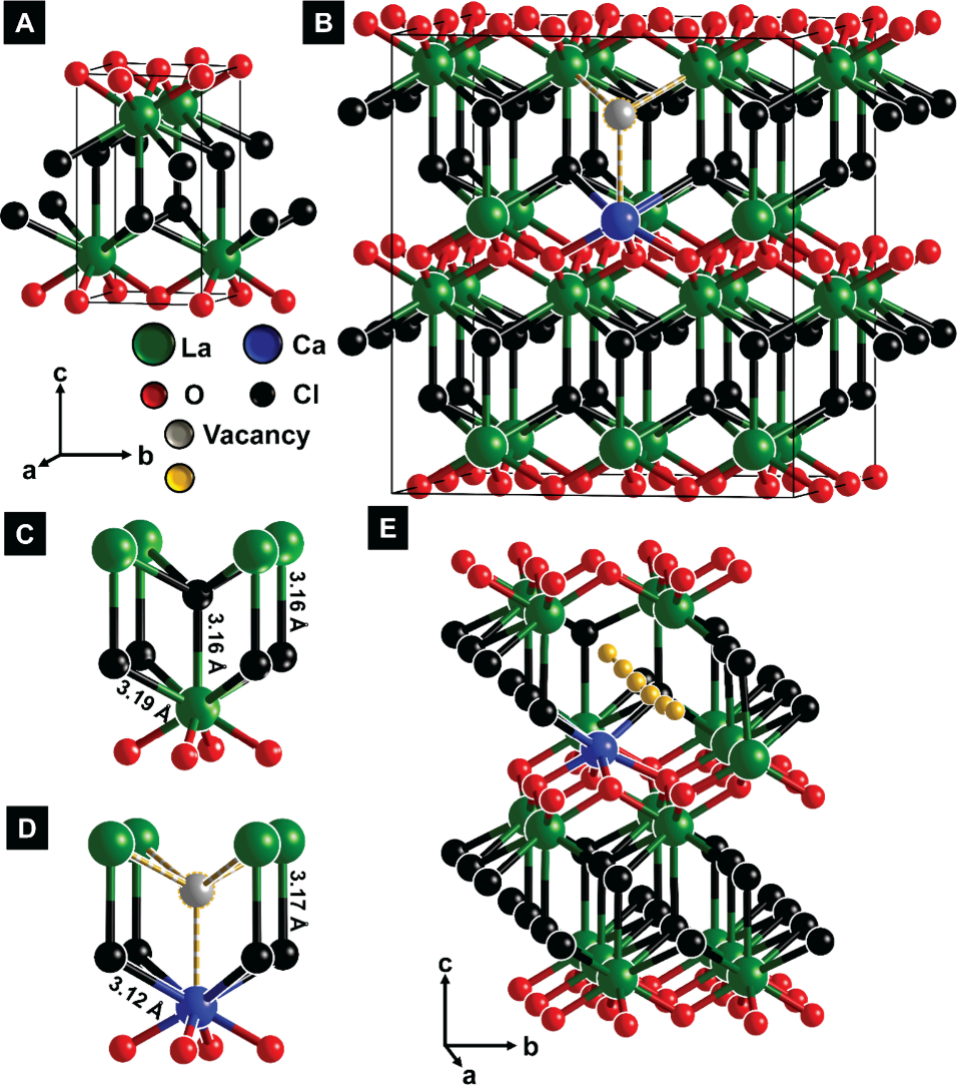
Point defects in LaOCl and implications of point defects for local structure. (A) Crystal structure of tetragonal LaOCl; (B) Ca-alloyed LaOCl with a chloride-ion vacancy shown in gray. La and Cl local coordination environments in (C) nondefective LaOCl and (D) Ca-alloyed LaOCl; the latter shows the modification of local structure induced upon aliovalent Ca-ion substitution, which is charge-balanced by introduction of a Cl-ion vacancy. (E) Migration pathway traced by a chloride-ion in a Ca-alloyed defective LaOCl crystal lattice as calculated from NEB simulations.

The local coordination environment of LaOCl is strongly modified by the alloying cations. For example, the nine-coordinated La^3+^ ion (with a Shannon-Prewitt radius of 1.356 Å)^56^ has La–Cl interatomic separations of ca. 3.19 and 3.16 Å in the lower and upper anion layers, respectively (Figure 1C). In contrast, based on geometry-optimized structures calculated using DFT, the alloyed Ca^2+^ ion with a Shannon–Prewitt radius of 1.26 Å^56^ is eight-coordinated and has a local coordination environment with Ca–Cl interatomic separations of ca. 3.23 Å, with an adjacent La–Cl bond length of ca. 3.20 Å to the upper anion layer (Figure 1D). The stronger Ca–O bond brings the substituent atom in closer proximity to the (LaO)^+^ layer while elongating adjacent Ca–Cl bonds. The Cl-ions adjacent to the chloride-vacancy introduced upon aliovalent Ca^2+^ substitution on the La sublattice are distorted further away from the vacancy. A nudged elastic band (NEB) calculation, shown in Figure 1E, reveals the most energetically favorable migration pathway for a lattice Cl-ion moving toward a neighboring vacancy site, consistent with a conventional Schottky-type ion conduction mechanism.^17^ Figure S1A–F contrast various short-range ion migration pathways and their associated energetics. In the single vacancy picture, ions migrate across the halide slabs through a trajectory that passes between the interplanar galleries of (LaO)^+^ and Cl^–^ layers. At higher vacancy concentrations, short-range clustering, divacancies, and correlated vacancy motion are expected to dominate, and may strongly influence transport behavior. Such divacancy or correlated cluster diffusion mechanisms require a more detailed examination of defect formation energy and defect level diagrams, as well as ab initio molecular dynamics simulations of ion migration across much larger supercells than considered here.^57^

Figure 2A,B display powder XRD patterns of Dy- and Tb-alloyed LaOCl nanocrystals with varying concentrations of Ca alloying (0–30 at. %). All XRD patterns are indexed to a tetragonal *P*4/*nmm* unit cell (*a* = *b* = 4.11 Å; *c* = 6.87 Å) consistent with the PbFCl structure type (ICSD 73-2063). These patterns indicate that Ca alloying nevertheless preserves the tetragonal PbFCl crystal structure, with only minimal shifts of the reflections. Rietveld refinements of the powder XRD patterns reveal that the unit cell volume contracts with increasing Ca (and Cl-ion vacancy) concentration (Figure S2); the *c* lattice separation shrinks more than the *a,b* lattice parameters as a result of the lower charge of Ca-ions and its smaller ionic radii.^29^ The Rietveld refinements to powder XRD patterns are plotted in Figures S3 and S4; refinement parameters, atom positions, and thermal parameters are listed in Table S2A–J.

**Figure 2:**
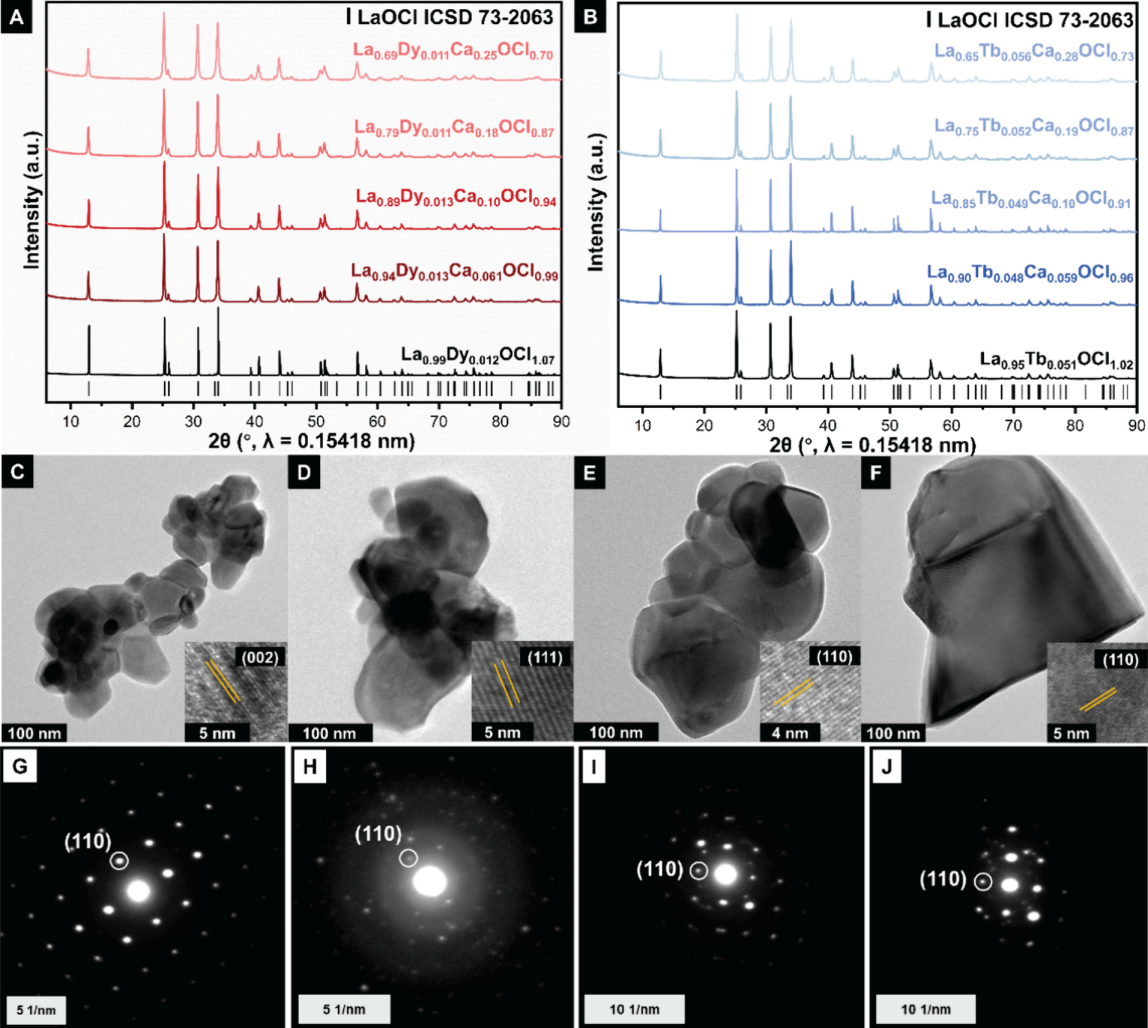
Structural characterization of alloyed LaOCl particles. Powder XRD pattern of (A) Dy-activated LaOCl with 0–30 at. % Ca alloying and (B) Tb-activated LaOCl with 0–30 at. % Ca alloying. Rietveld refinements and structure representations of the highest alloy concentrations are shown in Figures S3 and S4. Transmission electron microscopy images of (C) La_9.9_Dy_0.012_OCl_1.07_ particles; the HRTEM image in the inset shows the separation between (002) planes; (D) La_0.89_Dy_0.013_Ca_0.10_OCl_0.94_ particles; HRTEM image in the inset shows the separation between (111) planes; (E) La_0.99_Tb_0.012_OCl_1.02_ particles; HRTEM image in the inset shows the separation between (110) planes; (F) La_8.9_Tb_0.013_Ca_0.10_OCl_0.91_ particles; HRTEM image in the inset shows the separation between (110) planes; indexed SAED patterns of (G) La_9.9_Dy_0.012_OCl_1.07_; (H) La_8.9_Dy_0.013_Ca_0.10_OCl_0.94_; (I) La_9.9_Tb_0.012_OCl_1.02_; and (J) La_8.9_Tb_0.013_Ca_0.10_OCl_0.91_. SAED patterns have been indexed to ICSD 73-2063.

Figure 2C–F show TEM images of La_8.9_Dy_0.013_OCl_0.94_, La_8.9_Dy_0.013_Ca_0.10_OCl_0.94_, La_9.9_Tb_0.012_OCl_1.02_, and La_8.9_Tb_0.013_Ca_0.10_OCl_0.91_ particles, which adopt a nanoplatelet morphology and are clustered in the form of polygonal agglomerates. TEM analysis shows dimensions of about 80 ± 23 nm; no significant differences in particle dimensions are observed with increasing Ca-ion alloying (Figure S5); however, a slight preference for platelet morphologies with *ab* basal planes is noted, which may be attributed to the influence of Ca-alloying. SAED patterns and lattice-resolved HRTEM images in Figure 2C–F are consistent with the phase indexing to the PbFCl-structure-type (Figures S3, S4, and Table S2A–J). As such, the heating profiles selected during synthesis ensure that particles of similar dimensions can be compared with or without alloying.

### X-ray Probes of Defect Structure in LaOCl

To probe local structural distortions, we employ X-ray excited optical luminescence (XEOL) spectroscopy as a sensitive probe of halide-ion defects in LaOCl lattices alloyed with Tb and Dy chromophores on the La sublattice. In this approach, soft X-rays excite La core levels, generating “hot” electron–hole pairs.^33^ The absorbed energy is dissipated through two primary channels: (i) nonradiative Auger decay, in which high-energy electrons are ejected from the lattice; and (ii) inelastic phonon-mediated thermalization processes, which can transfer energy to nearby Dy and Tb luminescent centers.^58–61^ In the latter process, as excited electrons fall below the ionization threshold, electrons and holes thermalize via intraband transitions and electron–phonon scattering, eventually decaying to the conduction-band and midgap trap states from which they can participate in radiative recombination processes.^61,62^

The introduction of point defects such as halide vacancies significantly modifies these relaxation pathways. Vacancies act as transient electron traps, which can stabilize emitted Auger electrons in motifs reminiscent of electrides. These trapped carriers alter the balance between radiative and nonradiative channels, enabling broad-band emission associated with midgap defect states in addition to the sharp Dy^3^^+^ or Tb^3^^+^ *f–f* transitions. The broadness of the trap state emissions are a result of the variations in charge transition levels of different possible chloride vacancy configurations in the defect energy level diagram.^57,63^ As such, in defective lattices such as considered in this work, XEOL emission reflects both direct sensitization of lanthanide dopants via phonon-mediated inelastic processes and defect-mediated recombination through midgap states.^63^

Upon irradiation with soft X-rays, core-level excitation of the La cation sublattice is the primary absorption mechanism, as illustrated in Figure 3A,B. Phonon-mediated inelastic processes result in energy transfer to luminescent Dy and Tb centers, and activate subsequent radiative recombination channels, which are observed as optical emission.^64,65^ The introduction of point defects such as anion vacancies further provides a means of trapping emitted Auger electrons generated by core-level excitation (Figure 3A). The optical emission of “reporter” Tb/Dy species and the excitation-energy-dependent evolution of Auger decay processes provides detailed insight into local coordination environments, defect concentrations, and defect types.^32,62,66–68^ Compared to an unalloyed material without extrinsic defects, the interplay between Auger emission, defect trapping in midgap trap states, and Dy-sensitized recombination expands the diversity of relaxation pathways. The presence of halide vacancies thus explains both the suppression of Dy-centered emission at the La giant resonance (as a result of enhanced Auger emission losses) and the emergence of vacancy-related broad-band luminescence at high defect concentrations. A schematic energy transfer diagram (Figure 3A,B) illustrates these processes, highlighting the competing pathways of (i) phonon-mediated Dy sensitization, (ii) Auger-driven nonradiative losses, and (iii) Dy-sensitized defect-state and *f–f* emission.

**Figure 3:**
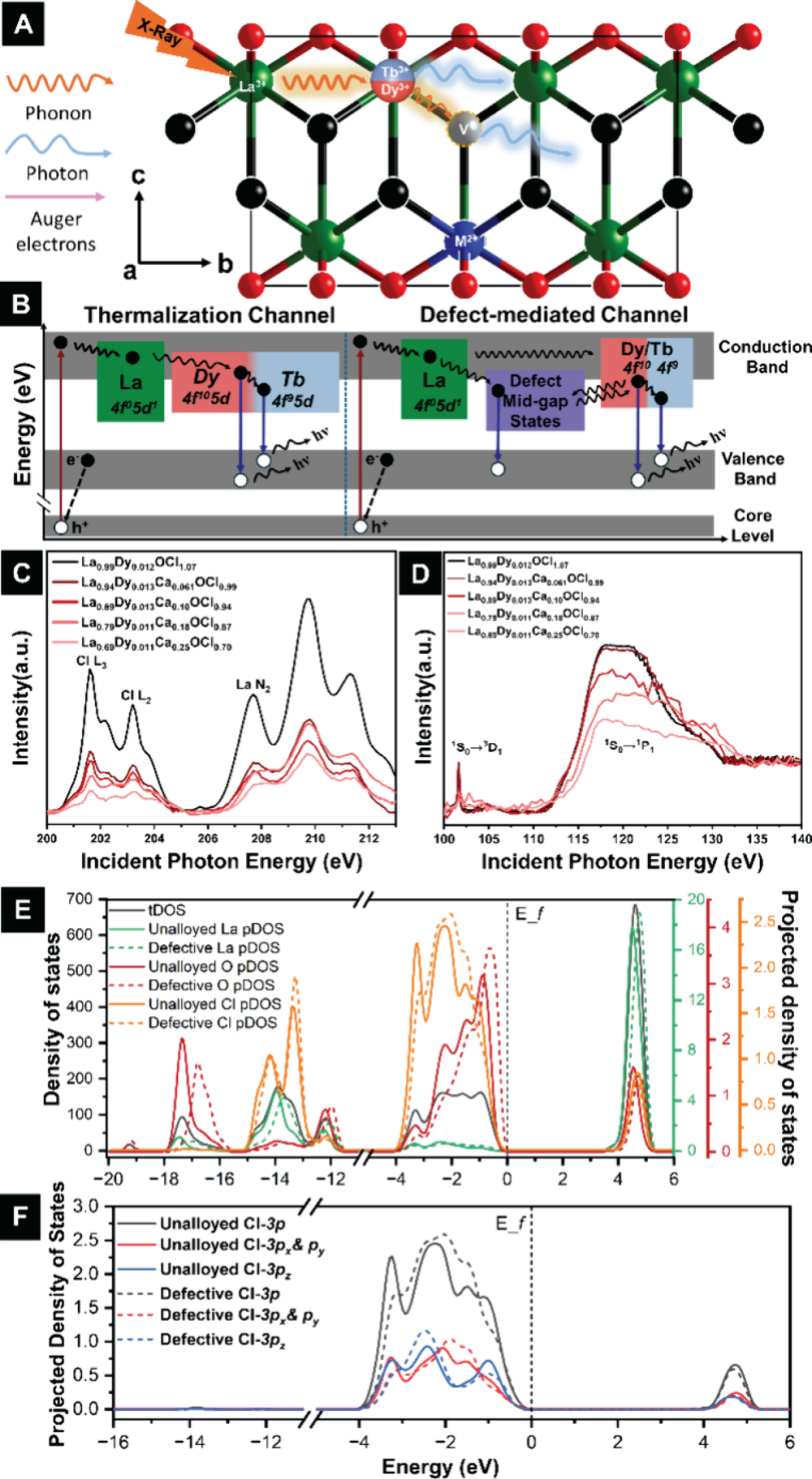
(A) Soft X-ray sensitization mechanism in Dy/Tb-alloyed defective LaOCl, showing La absorption, Auger emission, vacancy trapping, energy transfer to Dy/Tb, and radiative relaxation. (B) Corresponding energy-level schematic. (C) Cl L_2,3_ and La N_2_ XANES of La_1–*x*–*y*_Ca_*x*_Dy_*y*_OCl_1–*x*_. (D) La N_4,5_ edge XANES of La_1–*x*–*y*_Ca_*x*_Dy_*y*_OCl_1–*x*_. (E) Total DOS for unalloyed LaOCl and ca. 3.8 at. % Ca-alloyed La_0.96_Ca_0.038_OCl_0.96_. (F) La- and Cl-projected DOS highlighting enhanced Cl 3*p* contributions upon Ca alloying.

Figure 3C plots Cl L_2,3_-edge and La N_2_-edge XANES spectra acquired for LaOCl particles with 0.011–0.013 at. % Dy with increasing amounts of Ca-alloying (which in turn is correlated with increasing concentration of halide vacancies, Table S1). The Cl L-edge spectra correspond to excitation from Cl 2*p*_1/2_ (L_2_-edge) and 2*p*_3/2_ (L_3_-edge) states to Cl 4*s* states hybridized with La states.^69–75^ The La manifold corresponds to excitation of La 4*p*_1/2_ to 5*d* states. Comparing XANES spectra across the series with pre- and postedge normalization, lower intensities are observed with increasing Ca-alloying as a result of reduced La and Cl stoichiometries. In LaOCl, the Cl L_2_ edge absorption is centered at ca. 202 eV, and the L_3_ edge absorption at ca. 204 eV, which is slightly higher than in alkali chlorides.^73^ The higher energy of these absorption features likely reflects the strong La–Cl bonding in LaOCl.

Figure 3D plots La N_4,5_-edge XANES spectra, which correspond to La 4*d* → 4*f* excitations, for solid-solution LaOCl platelets with 0.011–0.013 at. % Dy with increasing amounts of Ca-alloying in the energy range between 100–140 eV. Internal standards measured for Eu-alloyed LaOCl capped with different passivating ligands are plotted in Figure S6.^29^ La^3+^-ions have a full 4*d* subshell but empty 5*d* and 4*f* states, and as such, are characterized by a singlet ^1^S_0_ ground state.^29,76^ The features observed in the XANES spectra in Figure 3D are assigned to 4*d* → 4*f* transitions from (i) the La ^1^S_0_ to a triplet ^3^D_1_ final state at 102 eV (which is formally forbidden, but observed as a result of spin–orbit coupling), and (ii) from the La ^1^S_0_ singlet state to a singlet ^1^P_1_ state at 120 eV (permitted by LS selection rules). Because of the strong overlap between La 4*d*- and 4*f*-derived bands in the electronic structure of LaOCl, an intense asymmetric giant resonance feature is observed centered at ca. 120 eV (which is ca. 10 eV above the ionization threshold). The broad line shape of giant resonance absorption reflects its short lifetime.^77^ Given that Dy is incorporated at a low concentration of 0.011–0.013 at. %, giant resonances characteristic of Dy luminescent centers are not visible in the XANES spectra. Interpretation of XANES spectra is aided by first-principles density functional theory (DFT) calculations of the total density of states (tDOS), which are shown in Figure 3E, along with atom-projected partial DOS (pDOS) in Figure 3F for the unalloyed lattice and the defective structure corresponding to La_0.98_Ca_0.02_OCl_0.98_ (Figure 1C,D). In both structures, the valence band (VB) primarily comprises Cl and O states, whereas La states dominate the conduction band (CB).^78^ In calculated DOS plots for the defective structure, anion defect states appear at higher energies, closer to the valence band. Due to the minimal changes in the conduction band (CB) states associated with La and Cl between the unalloyed and defective structures, the observed differences in the XANES spectra likely derives from other factors. One key contributor is local structural distortion caused by Cl vacancies, which alters the crystal field around La atoms and affects the final-state potential of the photoexcited electron. Additionally, charge redistribution due to vacancy-induced electron density imbalances can influence both the position and intensity of XANES features. While the valence band (VB) evolution does not directly explain the XANES features, it does shed light on the influence of vacancies on the local electronic structure. Specifically, the introduction of vacancies induces slight lattice distortions, which in turn shift valence states toward higher energies—an effect that reflects the perturbation of the local bonding environment.

Figure 4A,B plot 3D contour XEOL maps for unalloyed La_0.99_Dy_0.012_OCl_1.07_ and defective La_0.69_Dy_0.011_Ca_0.25_OCl_0.70_, which illustrate modulation of optical emission intensities upon excitation at different photon energies across the La N_4,5_-edge (corresponding XANES spectra are shown in Figure 3D). The plots thus illustrate the efficacy of phonon-mediated sensitization of the Dy centers and the radiative recombination channels accessible to the Dy centers upon 4*d* → 4*f* excitation of La^3+^ cations. 3D contour maps acquired for LaOCl platelets with intermediate amounts of Ca alloying are shown in Figure S7A–C. For both La_0.99_Dy_0.012_OCl_1.07_ and La_0.69_Dy_0.011_Ca_0.25_OCl_0.70_, it is apparent that the intensity of the optical luminescence features is greatly diminished upon excitation at the giant resonance centered at 117 eV in comparison to higher and lower energies. The observed diminution in intensity reflects activation of nonradiative recombination channels upon X-ray excitation at the La giant resonance.^29,39,79^ Indeed, such a suppression of radiative relaxation channels has been noted previously for Eu-doped LaOCl^76^ and NaLa_1–*x*_Dy_*x*_(MoO_4_)_2._ ^80^ In Figure 4A, the diminution of the optical luminescence as a function of incident photon energy is observed across a relatively narrow range of incident photon energies (117–120 eV) for La_0.99_Dy_0.012_OCl_1.07_, whereas in Figure 4B, for La_0.69_Dy_0.011_Ca_0.25_OCl_0.70_, the sample with the highest concentration of halide vacancies, a much more protracted regime of suppression of optical luminescence is observed between 115 and 123 eV. In general, Figure S8A–C illustrates that the range wherein nonradiative relaxation processes are dominant is successively expanded with increasing concentration of halide vacancies.

**Figure 4:**
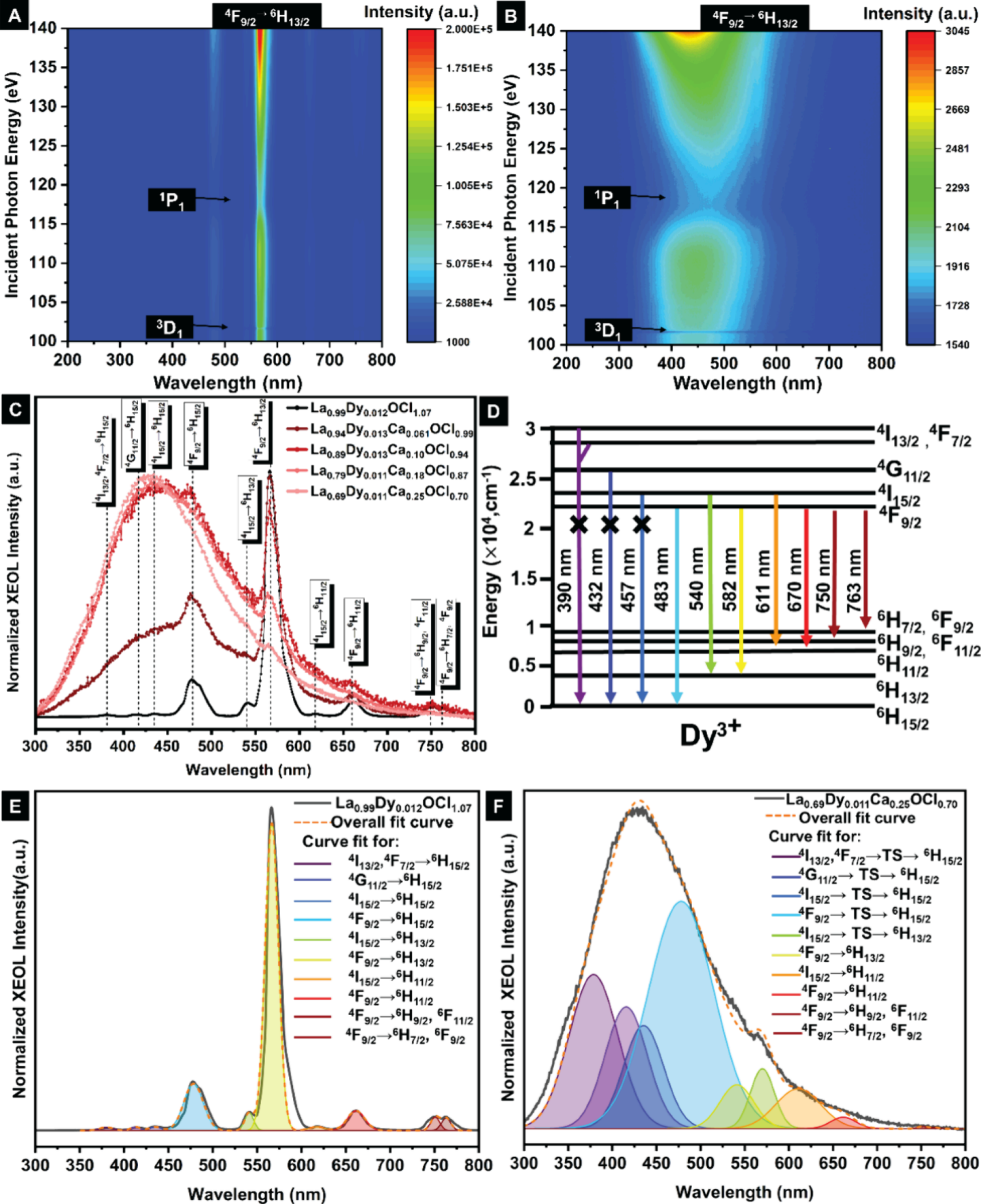
3D contour maps of XEOL signals as a function of incident photon energy for (A) unalloyed La_0.99_Dy_0.012_OCl_1.07_; (B) 30 at. % defects La_0.69_Dy_0.011_Ca_0.25_OCl_0.70_; XEOL profiles of LaOCl particles. (C) XEOL spectra of Dy-alloyed defective La_1–*x*–*y*_Ca_*x*_Dy_*y*_OCl_1–*x*_; (D) Dieke diagram illustrating sensitized emissions; fwhm curve fit of XEOL spectra for (E) La_0.99_Dy_0.012_OCl_1.07_ and (F) La_0.69_Dy_0.011_Ca_0.25_OCl_0.70_.

The predominant nonradiative channel at the giant resonance involves the emission of Auger electrons.^29,81^ With increasing concentration of halide vacancies, the lineshapes of the giant resonance are modified, which reflects an expanded diversity of La local coordination environments (Figure 3B). Auger emission is enhanced by the strong local potentials at halide vacancy sites and their ability to trap emitted electrons as transient electrides. Most notably, the introduction of halide vacancies within the crystal lattice weakens phonon-mediated La → Dy sensitization mechanisms, which suppresses the activation of Dy-centered luminescent emission. As such, the Auger emission signatures upon X-ray absorption at the La N-edge provide a sensitive measure of distortion of the La local coordination environment and the vacancy-induced diminution of accessible phonon relaxation pathways resulting from increasing concentrations of halide vacancies. Unlike Eu^3^^+^, which is well-known to undergo beam-induced reduction to Eu^2^^+^ under high-energy synchrotron irradiation owing to its readily accessible redox potential (Eu^3+^/Eu^2+^, – 0.92 V versus Ag/AgCl),^82^ Dy^3^^+^ and Tb^3^^+^ are more likely to preserve their formal valence especially under UHV conditions;^83–85^ redox potentials for Dy^3^^+^/Dy^2+^ and Tb^3^^+^/Tb^2+^ are −1.15 and −1.19 V,^82,86–88^ vs Ag/AgCl,^82^ respectively.^84,85^ Notably, no characteristic emission features of divalent lanthanides were detected in XEOL spectra acquired for Dy- or Tb-alloyed LaOCl up to chromophore concentrations of 1 at. % Dy and 5 at. % Tb.^83,89^

Below or above the giant resonance, nonresonant core-level excitation of La^3+^ generates multiple “hot” electron–hole pairs.^81,90^ Energy transfer to Dy dopants and thermalization activates multiple radiative recombination channels observed as emission bands in the visible region of the electromagnetic spectrum derived from both intraconfigurational 4*f–*4*f* transitions as well as transitions from midgap trap-states. Figure 4C plots XEOL spectra of LaOCl platelets doped with 0.011–0.013 at. % Dy with increasing amounts of Ca-alloying corresponding to X-ray excitation from 4*d*_3/2_ (N_4_-edge) to the 4*f* levels of La^3+^, for samples with 0–30 at. % Ca alloying concentrations. The corresponding Dieke diagram for Dy^3+^ energy manifolds is shown in Figure 4D and has been used to assign the emission bands in Figures 4 and S3A–C.^91^ Because the emergent bands are unusually broad, they cannot be attributed solely to Dy^3^^+^ *f–f* transitions, which typically maintain narrow line widths even at elevated temperatures.^92–94^ The observed line widths for highest Ca-ion alloying here is on the order of 4500 cm^–1^ or 0.5 eV fwhm, which exceeds even the 3000–3500 cm^–1^ line widths characteristic of *f*–*d* transitions of Ce^3+^. Instead, these features likely comprise contributions from defect-related trap states in addition to Dy^3^^+^ *f–f* emission. Prior studies on pristine rare-earth oxyhalides^95^ and BiOCl^96–98^ have linked such broadening to oxygen–chloride divacancies and their influence on the emission manifold. However, divacancies do not entirely account for our observations: comparable broadening is absent in the Tb-alloyed series and in previously examined Eu^2^^+^/Eu^3+^-alloyed samples.^76^ Figure 4E,F contrast distinctive emission spectra with peak fits of prominent emission bands for La_0.99_Dy_0.012_OCl_1.07_ without Ca-alloying (corresponding to the lowest concentration of halide vacancies) and La_0.69_Dy_0.011_Ca_0.25_OCl_0.70_, the most defective structure of the series with 30 at. % aliovalent Ca alloying on the La sublattice and the highest concentration of halide vacancies. fwhm curve fits for additional samples, La_0.94_Dy_0.013_Ca_0.061_OCl_0.99_, La_0.89_Dy_0.013_Ca_0.10_OCl_0.94_, and La_0.79_Dy_0.011_Ca_0.18_OCl_0.87_, are shown in Figure S8A–C. We classify channels below 560 nm as “blue channels” and these are observed to be greatly broadened with increased concentration of halide vacancies.^32,62,66–68^ As such, the broad emission features arise predominantly from defect-mediated trap-states as sensitized by Dy^3^^+^ ions (Figure 3B). The broadness of the band reflects a short lifetime and a diverse range of defect energy levels deriving from different vacancy atomic configurations. Specifically, defect-related midgap states reflect a continuum of localized states arising from a multitude of chloride vacancies as well as chloride–oxide vacancy clusters.^96^ In contrast, channels above 560 nm (“red channels”) exhibit nearly constant line widths and are consistently attributable to sharp thermalized Dy^3^^+^ *f–f* transitions.

Based on the Dieke diagram, the blue bands reflect emissions associated with thermally populated ^4^I_13/2_,^4^F_7/2,_ and ^4^G_11/2_ states as well as defect trap states, whereas the redder channels reflect for the most part emission from thermalized ^4^F_9/2_ states. Figure 4B,F show that for unalloyed La_0.99_Dy_0.012_OCl_1.07_, “hot” bands are significantly muted and the Dy ^4^F_9/2_ → ^6^H_13/2_ transition at 582 nm is the most prominent feature. Accordingly, the observed XEOL can be understood as the superposition of sharp Dy^3^^+^ *f–f* emission lines that dominate the red region (>560 nm) and broad, defect-mediated bands in the blue (<560 nm), whose prominence increases with halide-vacancy concentration. This framework underscores the central role of vacancy defects in governing recombination pathways, with Dy^3^^+^ ions functioning both as direct emitters and as sensitizers that couple excitation into midgap trap states when Dy^3^^+^ ions are in the proximity of halide vacancies. Figure S9 displays the CIE coordinates and their evolution with increasing concentration of halide vacancies.

Figure 5 plots Cl L_2,3_ and La N-edge XANES spectra for Tb-alloyed samples as well as XEOL 3D contour plots acquired upon excitation across the La N_4_-edges as well as a Dieke diagram for Tb. The Cl L_2,3_ XANES spectra closely resemble those for Dy-alloyed samples. Higher defect concentrations decrease the intensity of the Cl L- and La N-edge features; a pronounced broadening of Cl L_2,3_ edges is observed toward higher energies with increasing concentration of halide vacancies (Table S1), which reflects an increasingly diverse local structure and coordination environment for Cl-ions in the defective Cl-slabs of LaOCl (Figure 5A).

**Figure 5:**
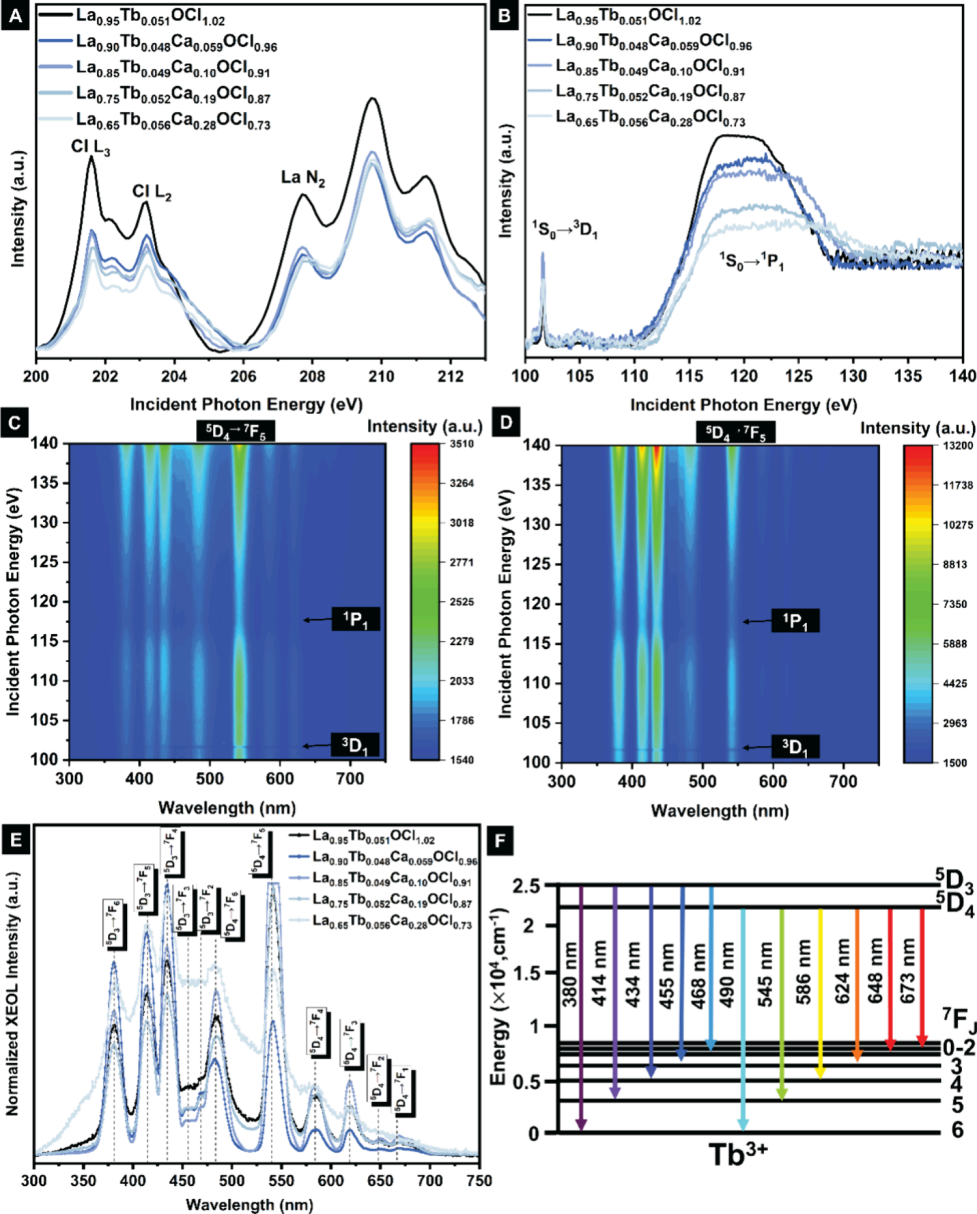
(A) Cl L_2,3_ and La N_2_ edge XANES spectra of La_1–*x*–*y*_Ca_*x*_Tb_*y*_OCl_1–*x*_; (B) La N_4,5_ edge XANES spectra of La_1–*x*–*y*_Ca_*x*_Tb_*y*_OCl_1–*x*_; 3D contour maps of XEOL signals as a function of incident photon energy for (C) unalloyed La_0.99_Tb_0.051_OCl_1.02_; (D) 27 at. % defects La_0.69_Tb_0.056_Ca_0.28_OCl_0.73_; (E) XEOL spectra of Dy-activated defective La_1–*x*–*y*_Ca_*x*_Tb_*y*_OCl_1–*x*_; (F) Dieke diagram illustrating sensitized emissions.

3D contour XEOL maps acquired for unalloyed La_0.99_Tb_0.051_OCl_1.02_ and the most defective structure upon Ca alloying (La_0.69_Tb_0.056_Ca_0.28_OCl_0.73_) are contrasted in Figure 5C,D. Figure 5E plots the XEOL response for LaOCl platelets doped with 0.049–0.056 at. % Tb with increasing amounts of Ca-alloying corresponding to X-ray excitation at 4*d*_3/2_ (N_4_-edge) to the 4*f* levels of La^3+^, for samples with 0–30 at. % Ca alloying concentrations. Figure 5F shows the relevant Dieke diagram. Figure S5A,E contrast distinctive emission spectra with peak fits of prominent emission bands for La_0.99_Tb_0.051_OCl_1.02_ without Ca-alloying (corresponding to the lowest concentration of halide vacancies) and La_0.69_Tb_0.056_Ca_0.28_OCl_0.73_, the most defective structure of the series with 30 at. % aliovalent Ca alloying on the La sublattice and the highest concentration of halide vacancies. The following radiative relaxation channels are observed: 380 nm (^5^D_3_ → ^7^F_6_), 414 nm (^5^D_3_ → ^7^F_5_), 434 nm (^5^D_3_ → ^7^F_4_), 455 nm (^5^D_3_ → ^7^F_3_), 468 nm (^5^D_3_ → ^7^F_2_), 490 nm (^5^D_4_ → ^7^F_6_), 545 nm (^5^D_4_ → ^7^F_5_), 586 nm (^5^D_4_ → ^7^F_4_), 624 nm (^5^D_4_ → ^7^F_3_), 648 nm (^5^D_4_ → ^7^F_2_), and 673 nm (^5^D_4_ → ^7^F_1_).^99^ A similar suppression of optical luminescence is observed upon excitation across the La giant resonance in the energy range of 117–120 eV as for the Dy-alloyed samples. The diminished optical luminescence similarly corresponds to the activation of nonradiative Auger emission channels but for Tb-alloyed samples, the nonradiative range is not substantively expanded upon introduction of halide vacancies, which in turn reflects the strong sensitization of Tb centers upon X-ray excitation of La core levels.^29,76^ With increasing concentration of halide vacancies, broader emission bands are observed further reflecting activation of defect-mediated trap states as well as a pronounced perturbation and extensive variations of the local coordination environment of Tb^3+^ ions upon structure modification and introduction of halide vacancies. An increase in the intensity of “blue channels” below 468 nm is observed as compared to “red channels” in the wavelength range of 490–673 nm. While for the unalloyed La_0.99_Tb_0.051_OCl_1.02_ structure, Figures S5E and 5D show that the most prominent emission corresponds to the 490 nm band derived from ^5^D_4_ → ^7^F_6_ relaxation, introduction of halide vacancies such as for La_0.69_Tb_0.056_Ca_0.28_OCl_0.73_ greatly enhances the blue emission bands at 468 and 434 nm derived from ^5^D_3_ → ^7^F_2_ and ^5^D_3_ → ^7^F_4_ transitions, respectively. fwhm analysis for the intermediate Ca-alloying concentrations are shown in Figure S10A–E. Their respective 3D XANES-XEOL contour maps are shown in Figure S11A–C. Peaks are categorized into “blue” and “red” channels, denoting wavelengths below and above 468 nm, respectively. Upon the introduction of halide vacancies, radiative relaxation is greatly enhanced from thermally populated ^5^D_3_ states as compared to ^5^D_4_ states, suggesting that phonon-mediated thermalization pathways are greatly reduced with increasing concentration of point defects. Figure S12 shows the CIE coordinates for Tb-alloyed samples upon X-ray excitation.

Considering Figure 3A,B, in the case of Dy/Tb-alloyed LaOCl, we observe nonradiative Auger electron emission at the giant resonance. With increasing halide vacancy concentration, the giant resonance is expanded because chloride vacancies serve as trap states that stabilize Auger electrons as transient electrides. The presence of anion vacancies disrupts phonon-mediated La → Dy sensitization, thereby enhancing nonradiative recombination.^59^ In the case where Tb/Dy atoms are substitutionally alloyed on La sites in LaOCl in the immediate proximity of halide vacancies, strong trap state emission is observed in addition to intraconfigurational *f–f* transitions. New radiative relaxation channels are activated by the presence of trap-related midgap states for Dy^3+^ chromophores, which give rise to broad-band luminescence in the blue region. Because trapping bypasses full phonon thermalization, the resulting emission originates from nonthermalized states and is significantly enhanced with increasing halide vacancy concentration. Conversely, where Dy/Tb centers are not in the immediate proximity of halide vacancies, carriers thermalize through phonon scattering in and radiative recombination proceeds via well-defined *f–f* (Dy) or *f–d* (Tb) transitions from Dy^3+^/Tb^3+^ states albeit with considerable modification of lineshapes, which reflects considerable perturbation of the local coordination environment with increasing incorporation of aliovalent Ca-ions and charge-compensating chloride vacancies.^29,76,90^ Taken together, the relative contributions of (i) Auger electron losses stabilized at vacancy traps, (ii) defect-mediated radiative recombination channels activated by chloride vacancies, and (iii) phonon-mediated radiative recombination from thermalized states provide direct insight into the concentration and distribution of halide vacancies in LaOCl.

We have also measured the excitation and emission spectra of pristine Dy-alloyed (La_0.99_Dy_0.012_OCl_1.07_) sample through a laboratory spectrophotometer, where we observe similar bands for Dy^3+^ in Figure S13. However, emission yields for this wide bandgap (estimated to be ca. 5.54 eV) material are orders of magnitude lower upon UV–visible (below bandgap) excitation as compared to element/edge-specific core-level excitation with monochromatic soft X-rays.

### Chloride-Ion Conduction in LaOCl

Figure 6A,B plots temperature-dependent ion conductivity measured using electrochemical impedance spectroscopy (EIS) for pellets of LaOCl nanoplatelets with 0–30 at. % Ca substitution. The ionic conductivity values have been obtained by fitting the equivalent circuit for each EIS plot to extract the resistance, as demonstrated for La_0.90_Ca_0.08_OCl_0.92_ in Figure 6B. The ionic conductivity increases monotonically from 25 to 400 °C without discontinuous phase transformations such as observed for LaOF.^16,100^ In general, the ionic conductivity increases with increasing concentration of halide vacancies up to a plateau of ca. 20 at. % Ca alloying.^16,100^ The observed plateau at high vacancy concentrations likely derives from defect clustering and formation of divacancies.

**Figure 6:**
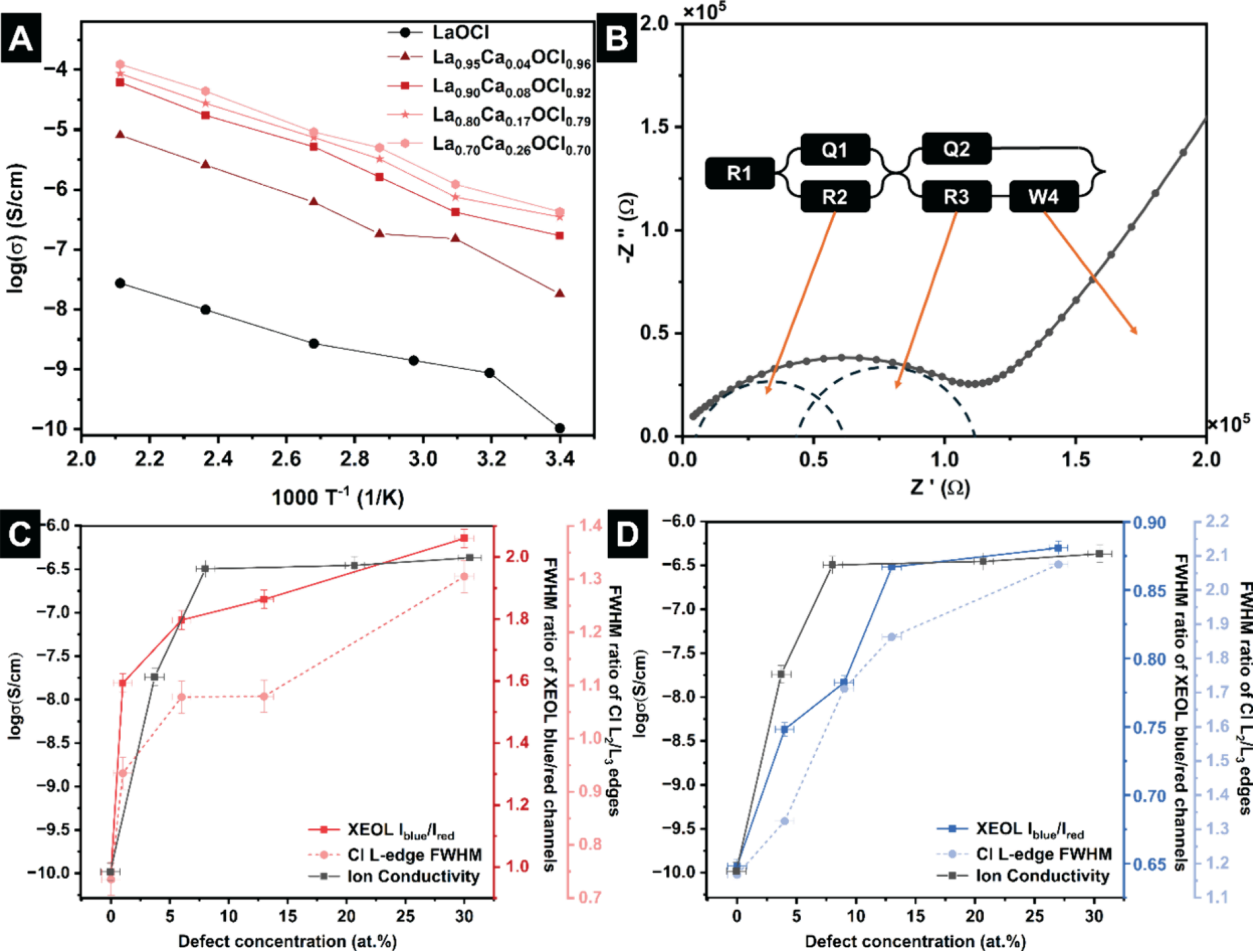
(A) Ion conductivity of La_1–*x*_Ca_*x*_OCl_1–*x*_ (*x* = 0–30 at. %) as a function of temperature from 25 to 400 °C; (B) example of equivalent circuit fitting for La_1–*x*_Ca_*x*_OCl_1–*x*_ at 25 °C and physical model of the equivalent circuit fitting with the resistance of grain and grain boundary and Warburg diffusion coefficient; correlation between defect concentration and fwhm ratio of XEOL blue and red channels (solid lines), defect concentration and fwhm ratio of XANES Cl L_2_ and L_3_ edges (dash lines) for (C) Dy- (red lines) and (D) Tb- (blue lines) activated samples, and defect concentration and ion conductivity (black solid lines).

To better understand the relationship between halide vacancy concentration, ionic conductivity, and the spectroscopic signatures, we examine the evolution of XEOL and XANES features as a function of point defect concentration as measured by NAA (Figure 6C,D). The fwhm of Cl L_2,3_-edge resonances provides an excellent measure of the diversity of coordination environments in the Cl-ion slab of LaOCl as illustrated in Figures S14, S15, Tables S3, and S4. With increasing concentration of halide vacancies, Figure 6C,D shows that the fwhm of Cl L-edge resonances is increased as a result of different local coordination environments for Cl-ions in highly distorted slabs.

We have further examined the relative intensities of “blue” and “red” channels (Tables S5 and S6) for Dy- and Tb-alloyed LaOCl platelets with 0–30 at. % vacancies. The crossover wavelength is defined as 560 nm for Dy-alloyed LaOCl and 468 nm for Tb-alloyed LaOCl to represent whether emission is dominated by thermally populated or thermalized states. Figure 6C,D illustrate that the integrated intensity of blue and red emission bands *I*_blue_/*I*_red_ serves is monotonically correlated with the halide vacancy concentration and maps directly to ion conductivity in LaOCl systems. While the XANES and optical luminescence signatures are well correlated with vacancy concentration, the ion conductivity starts to plateau at high vacancy concentrations. This plateauing is likely a result of vacancy clusters and correlated migration of defect clusters as short-range electrostatic interactions between point defects become increasingly prominent at high vacancy concentrations. As such, X-ray absorption spectroscopy signatures and XANES–XEOL maps provide detailed insight into defect concentrations and the evolution of phonon-mediated energy transfer pathways and ion conductivity with increasing concentration of chloride vacancies.

## Conclusions

In conclusion, site-selective aliovalent substitution of Ca-ions on the cation sublattice of LaOCl engenders halide vacancies in well-separated slabs of halide-ions. Ca-ion alloying and accompanying halide vacancies distort the local La and Cl coordination environments and facilitate vacancy migration along deformed halide layers, which have a phonon dispersion that is substantially modified from the intact lattice. Ionic conductivity in the range of 2.76 × 10^–5^–4.3 × 10^–5^ S/cm can be achieved at 300 °C at Cl vacancy concentrations of ca. 20 at. %, which holds promise for utilization as thermally robust and high-breakdown-voltage solid electrolytes of halide-ion batteries with minimal electronic conductivity.

Soft X-ray spectroscopy and XEOL measurements provide detailed insights into halide local coordination environments, distortion of local structure, and defect-mediated modification of phonon dispersion in defective LaOCl solid solutions, which in turn can be mapped directly to anion conductivity. It is important to highlight that Cl L-edge XANES remains largely underexplored in the literature. We demonstrate that Cl L-edge XANES, particularly in combination with complementary spectroscopic techniques such as XEOL, provides insight into the electronic structure of lanthanum oxychlorides. The fwhm of Cl L-edge XANES resonances is found to be correlated with the concentration of halide vacancies, reflective of distortions induced in the halide slab and the greater mobility of Cl-ions, which in turn begets a broad diversity of chloride local coordination environments in the crystal lattice. XEOL measurements reveal two distinct dissipative channels upon VUV excitation of La core-levels. On the one hand, excitation within the giant resonance results in the nonradiative emission of Auger electrons. Halide vacancies disrupt the phonon band structure and hinder thermalization mechanisms since phonon-mediated pathways are not accessible to enable efficient energy transfer, which thereby expands the energy range of the giant resonance. Such point defects can further trap emitted electrons to form transient electrides. On the other hand, excitation above and below the giant resonance can sensitize La → Dy/Tb energy transfer followed by activation of radiative recombination channels at the luminescent chromophores. The introduction of halide vacancies greatly intensifies blue emission from thermally populated or defect-related trap states associated with Dy^3+^/Tb^3+^ chromophores. The ratio of luminescence from thermally populated and trap states as compared to thermalized states provides a sensitive measure of phonon dispersion and maps with high fidelity to the concentration of halide vacancies and anion conductivity. The results demonstrate the sensitivity of La^3+^ core-level excitation and lattice coupled energy dissipation mechanisms to the vacancy concentration. It is noteworthy that the utility of this approach is limited at high defect concentrations given the formation of defect clusters and the increasingly prominent role of correlated motion of multivacancy domains.

The findings suggest a practicable route for tuning defect concentration and dopant incorporation to optimize ion conductivity while maintaining structural stability, which makes LaOCl solid solutions a promising candidate for solid electrolytes of Cl-ion and other anion battery systems. The results further demonstrate the pivotal role of defect engineering in modulating phonon-mediated energy- and ion-transfer processes. Future work will focus on largescale simulations of point defect interactions to examine how defect clusters modify anion mobility in these structures. Quasi-inelastic neutron scattering measurements in concert with large-scale ab initio molecular dynamics simulations for LaOCl with varying concentration of Cl-ion vacancies will help disentangle the role of single vacancy hoping and the correlated motion of defect clusters.

## Supplementary Material



## Notes

Supplementary figures and tables, refinement details, computational data and characterization data (PDF)
