# Supplementary material for: Defect Structure and Anion Conduction in Lanthanum Oxychloride Solid Solutions Revealed by X‑ray Excited Optical Luminescence and Auger Emission
Source: Chem Mater. 2025 Dec 3;37(24):9713–27. doi: 10.1021/acs.chemmater.5c01868 (PMC12746413; doi:10.1021/acs.chemmater.5c01868)
Supplement: Supplementary file 1 [file cm5c01868_si_001.pdf]

Defect Structure and Anion Conduction in Lanthanum Oxychloride Solid Solutions Revealed by X-ray Excited Optical Luminescence and Auger Emission

*Jingxiang Cheng,[1,2] Victor Alexander Gomez,[3,4] Jaime R. Ayala,[1,2] Alice R. Giem,[1,2] Arnab Maji,[1,2] Shruti Hariyani,[1,2,4] Lucia Zuin,[5] and Sarbajit Banerjee[2,3,4]**

[1]Department of Chemistry, Texas A&M University, College Station, TX 77843-3012, United States

[2] Department of Material Science and Engineering, Texas A&M University, College Station, TX 77843-3012, United States

[3]Laboratory for Inorganic Chemistry, Department of Chemistry and Applied Biosciences, ETH Zurich, Vladimir-Prelog-Weg 2, CH-8093 Zürich, Switzerland

[4] Laboratory for Battery Science, PSI Center for Energy and Environmental Sciences, Paul Scherrer Institute, Forschungsstrasse 111, CH-5232 Villigen PSI, Switzerland

[5] Canadian Light Source, University of Saskatchewan, Saskatoon, SK S7N 2V3, Canada

Correspondence: sbanerje@ethz.ch

Table S1. Mole ratio of all precursors used, expected, and actual product composition of Dy- and Tb-alloyed products.

Precursors (mole ratio)				Expected product composition	Actual product composition
yDy_2O_3	$(1-x-y)$ La_2O_3	$2x$ $(COO)_2Ca$	$(2-2x-2y)$ NH_4Cl	$La_{1-x-y}Dy_yCa_xOCl_{1-x}$	$La_{1-x-y}Dy_yCa_xOCl_{1-x}$
0.01	0.99	0	2	$La_{0.99}Dy_{0.01}OCl$	$La_{0.99}Dy_{0.012}OCl_{1.07}$
0.01	0.94	0.1	1.9	$La_{0.94}Dy_{0.01}Ca_{0.05}OCl_{0.95}$	$La_{0.94}Dy_{0.013}Ca_{0.061}OCl_{0.99}$
0.01	0.89	0.2	1.8	$La_{0.89}Dy_{0.01}Ca_{0.1}OCl_{0.9}$	$La_{0.89}Dy_{0.013}Ca_{0.10}OCl_{0.94}$
0.01	0.79	0.4	1.6	$La_{0.79}Dy_{0.01}Ca_{0.2}OCl_{0.8}$	$La_{0.79}Dy_{0.011}Ca_{0.18}OCl_{0.87}$
0.01	0.69	0.6	1.4	$La_{0.69}Dy_{0.01}Ca_{0.3}OCl_{0.7}$	$La_{0.69}Dy_{0.011}Ca_{0.25}OCl_{0.70}$
yTb_2O_3	$(1-x-y)$ La_2O_3	$2x$ $(COO)_2Ca$	$(2-2x-2y)$ NH_4Cl	$La_{1-x-y}Tb_yCa_xOCl_{1-x}$	$La_{1-x-y}Tb_yCa_xOCl_{1-x}$
0.05	0.95	0	2	$La_{0.95}Tb_{0.05}OCl$	$La_{0.95}Tb_{0.051}OCl_{1.02}$
0.05	0.9	0.1	1.9	$La_{0.9}Tb_{0.05}Ca_{0.05}OCl_{0.95}$	$La_{0.9}Tb_{0.048}Ca_{0.059}OCl_{0.96}$
0.05	0.85	0.2	1.8	$La_{0.85}Tb_{0.05}Ca_{0.1}OCl_{0.9}$	$La_{0.85}Tb_{0.049}Ca_{0.10}OCl_{0.91}$
0.05	0.75	0.4	1.6	$La_{0.75}Tb_{0.05}Ca_{0.2}OCl_{0.8}$	$La_{0.75}Tb_{0.052}Ca_{0.19}OCl_{0.87}$
0.05	0.65	0.6	1.4	$La_{0.65}Tb_{0.05}Ca_{0.3}OCl_{0.7}$	$La_{0.65}Tb_{0.056}Ca_{0.28}OCl_{0.73}$

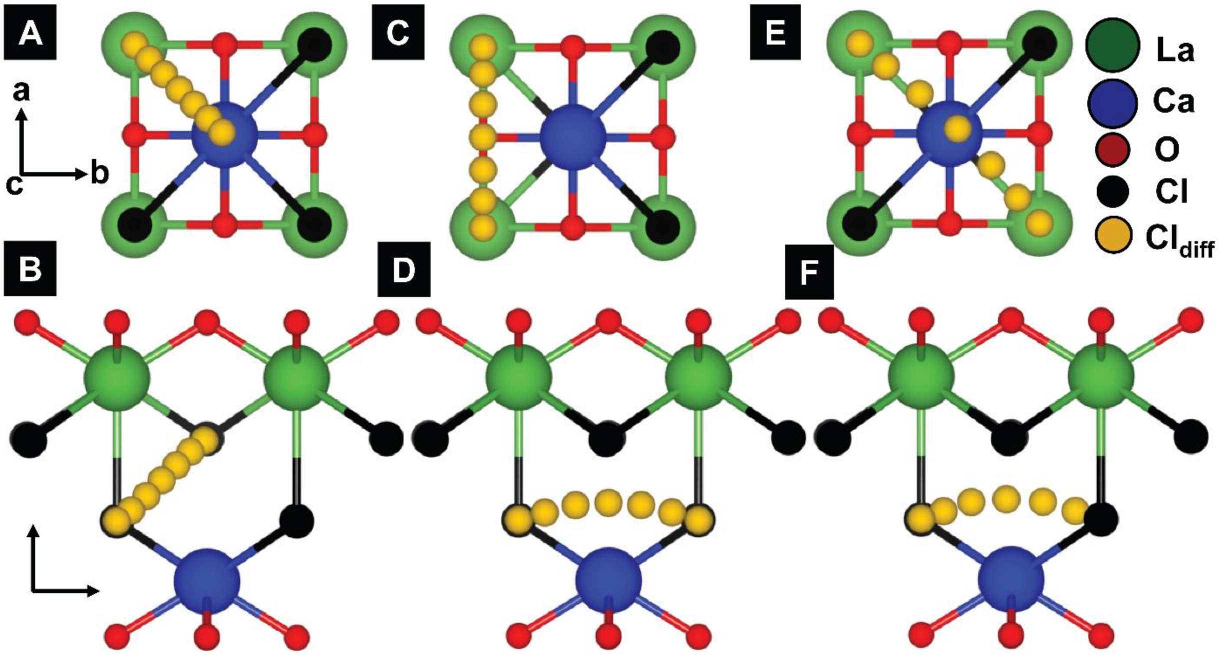

Figure S1. NEB calculations of three proposed Cl-ion migration pathways. A,B) *bc*-plane hopping (3.39 Å, 0.304 eV); C,D) *ab*-plane hopping (4.03Å, 0.627 eV); and E,F) diagonal *ab*-plane hopping (5.70 Å, 0.878 eV).

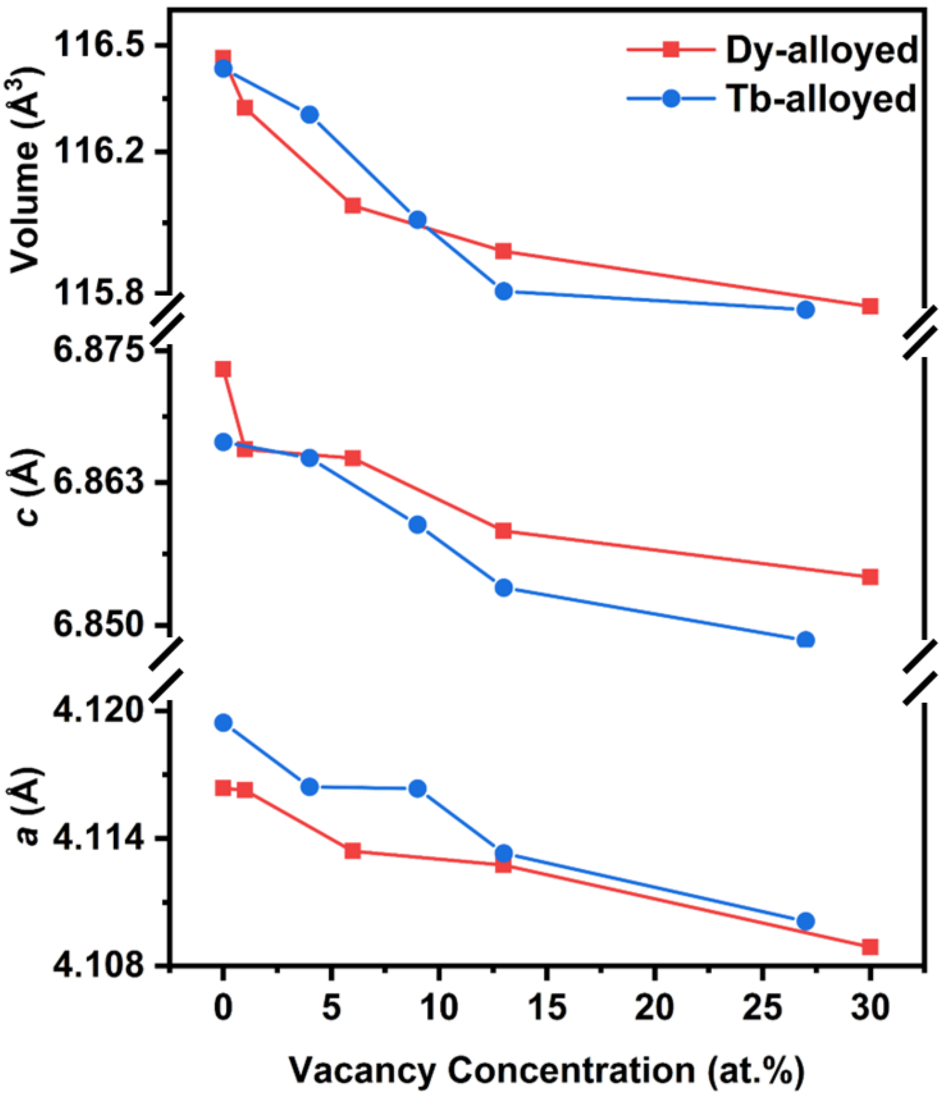

Figure S2. Evolution of lattice parameters and unit cell volume as derived from Rietveld refinements with increasing concentration of Ca-ions and chloride vacancies. The Tb and Dy concentrations are held constant at 5 and 1 at.%, respectively. The vacancy concentrations are inferred from NAA measurements.

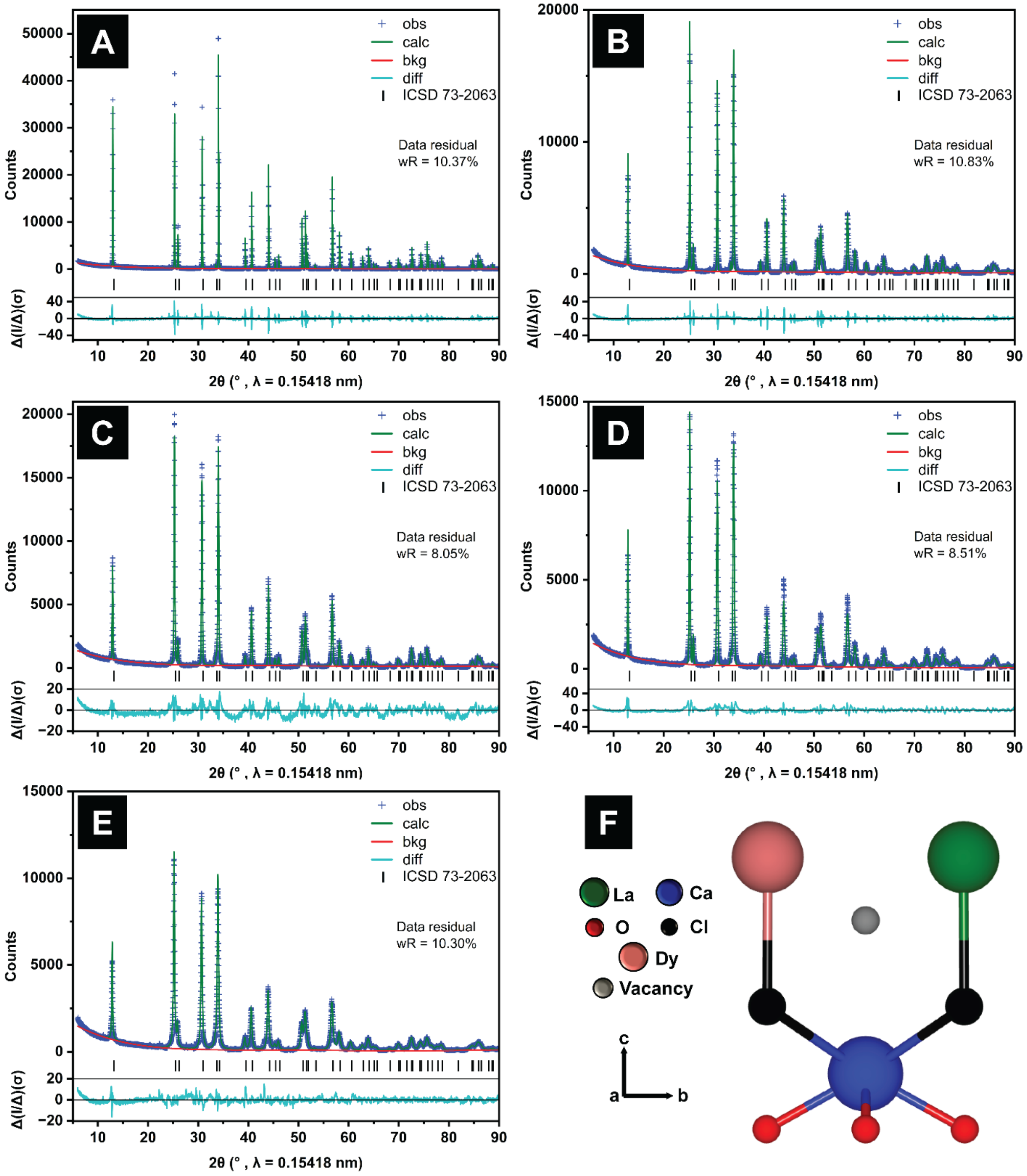

Figure S3. Rietveld refinements of powder XRD Patterns of (A) $La_{0.99}Dy_{0.012}OCl_{1.07}$; (B) $La_{0.94}Dy_{0.013}Ca_{0.061}OCl_{0.99}$; (C) $La_{0.89}Dy_{0.013}Ca_{0.10}OCl_{0.94}$; (D) $La_{0.79}Dy_{0.011}Ca_{0.18}OCl_{0.87}$; (E) $La_{0.69}Dy_{0.011}Ca_{0.25}OCl_{0.70}$ and (F) Refined crystal structure of $La_{0.69}Dy_{0.011}Ca_{0.25}OCl_{0.70}$.

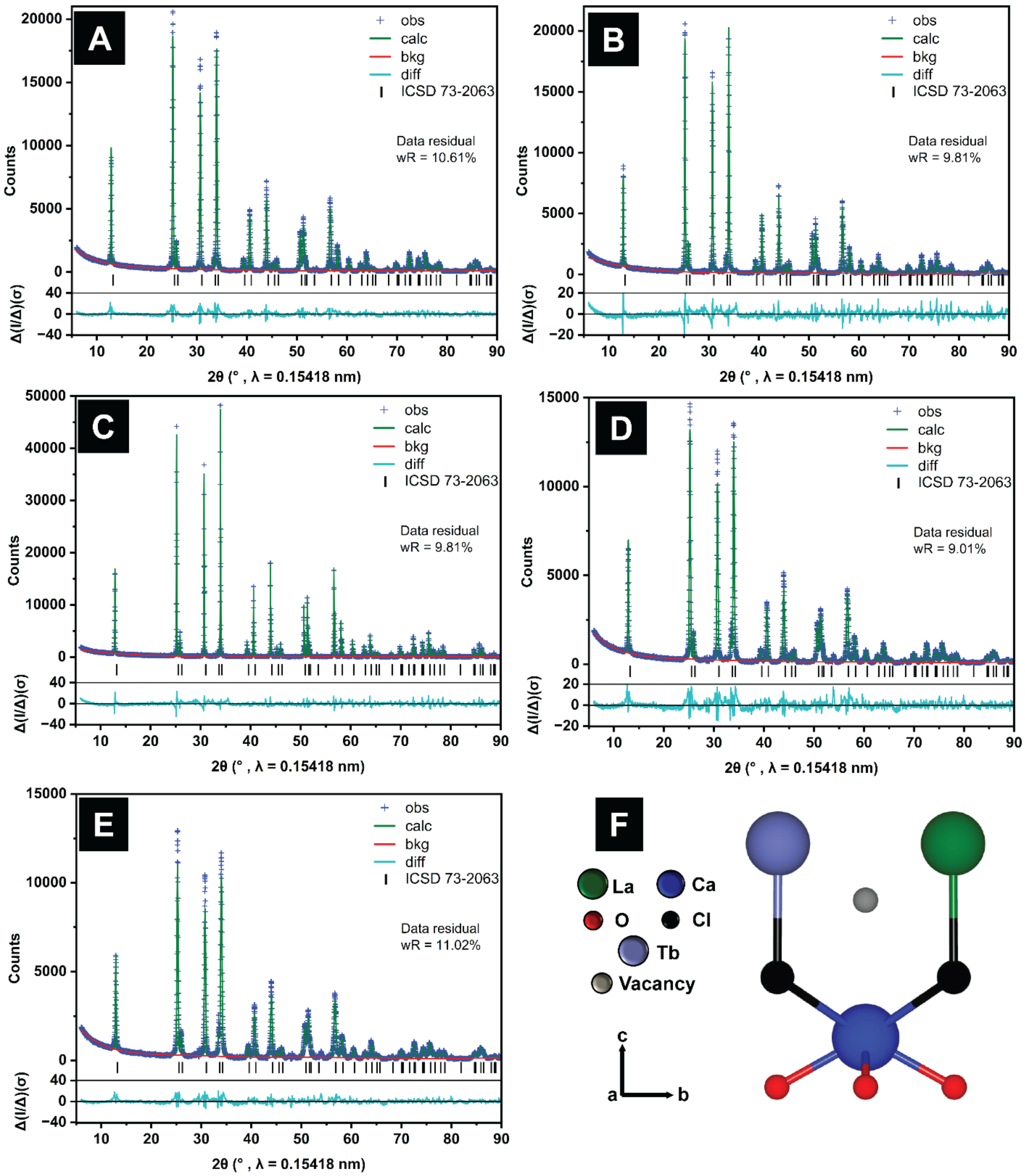

Figure S4. Rietveld refinements of powder XRD Patterns of (A) $La_{0.99}Tb_{0.051}OCl_{1.02}$; (B) $La_{0.9}Tb_{0.048}Ca_{0.059}OCl_{0.96}$; (C) $La_{0.85}Tb_{0.049}Ca_{0.10}OCl_{0.91}$; (D) $La_{0.75}Tb_{0.052}Ca_{0.19}OCl_{0.87}$; (E) $La_{0.69}Tb_{0.056}Ca_{0.28}OCl_{0.73}$ and (F) Refined crystal structure of $La_{0.69}Tb_{0.056}Ca_{0.28}OCl_{0.73}$.

Table S2A. Refined lattice parameters, atomic positions and thermal parameters for $La_{0.99}Dy_{0.012}OCl_{1.07}$.

**$La_{0.99}Dy_{0.012}OCl_{1.07}$**						
wR	10.37%	R	8.22%	χ^2	2.8	
2θ range	6 - 90°	Radiation	Cu Kα 1.5406 Å	Temp	295 K	
Formula	$La_{1-x-y}Dy_yCa_xOCl_{1-x}$	Z	2	V (Å³)	116.464	
a=b (Å)	4.1163(7)	*c (Å)*	6.8732(8)	S.G.	*P4/nmm*	
Atom	x	y	z	frac	Wyckoff	Uiso
Dy	0.0030(5)	0.4970(2)	0.1739(4)	0.990	2c	0.0080(5)
La	0.0015(1)	0.5022(3)	0.1756(8)	0.012	2c	0.0045(3)
O	0.9985(4)	0.0015(0)	0.0015(2)	1.000	2a	0.0130(6)
Cl	0.0010(7)	0.4985(6)	0.6321(6)	1.070	2c	0.0128(4)

Table S2B. Refined lattice parameters, atomic positions and thermal parameters for $La_{0.94}Dy_{0.013}Ca_{0.061}OCl_{0.99}$.

**$La_{0.94}Dy_{0.013}Ca_{0.061}OCl_{0.99}$**						
wR	10.83%	R	7.75%	χ^2	3.3	
2θ range	6 - 90°	Radiation	Cu Kα 1.5406 Å	Temp	295K	
Formula	$La_{1-x-y}Dy_yCa_xOCl_{1-x}$	Z	2	V (Å³)	116.324	
a=b (Å)	4.1162(7)	*c (Å)*	6.8660(2)	S.G.	*P4/nmm*	
Atom	x	y	z	frac	Wyckoff	Uiso
Dy	0.0031(1)	0.4969(9)	0.1740(8)	0.940	2c	0.0103(7)
La	0.0016(6)	0.5031(6)	0.1758(0)	0.013	2c	0.0048(9)
Ca	0.0012(9)	0.5026(3)	0.1752(2)	0.061	2c	0.0045(1)
O	0.9991(9)	0.0017(7)	0.0010(4)	1.000	2a	0.0136(1)
Cl	0.0012(8)	0.4979(4)	0.6330(6)	0.990	2c	0.0129(4)

Table S2C. Refined lattice parameters, atomic positions and thermal parameters for $La_{0.89}Dy_{0.013}Ca_{0.10}OCl_{0.94}$.

**$La_{0.89}Dy_{0.013}Ca_{0.10}OCl_{0.94}$**					
wR	8.05%	R	4.73%	χ^2	2.9
2θ range	6 - 90°	Radiation	Cu Kα 1.5406 Å	Temp	295K
Formula	$La_{1-x-y}Dy_yCa_xOCl_{1-x}$	Z	2	V (Å³)	116.048

a=b (Å)	4.1134(0)	*c (Å)*	6.8652(1)	S.G.	*P4/nmm*	
Atom	x	y	z	frac	Wyckoff	Uiso
Dy	0.0022(1)	0.4973(1)	0.1732(2)	0.890	2c	0.0111(9)
La	0.0015(9)	0.5018(2)	0.1751(7)	0.013	2c	0.0050(6)
Ca	0.0011(7)	0.5014(9)	0.1746(7)	0.100	2c	0.0046(8)
O	0.9983(3)	0.0019(1)	0.0009(7)	1.000	2a	0.0141(2)
Cl	0.0014(1)	0.4987(6)	0.6328(8)	0.940	2c	0.0120(2)

Table S2D. Refined lattice parameters, atomic positions and thermal parameters for $La_{0.79}Dy_{0.011}Ca_{0.18}OCl_{0.87}$.

$La_{0.79}Dy_{0.011}Ca_{0.18}OCl_{0.87}$						
wR	8.51%	R	5.74%	χ^2	2.7	
2θ range	6 - 90°	Radiation	Cu Kα 1.5406 Å	Temp	295K	
Formula	$La_{1-x-y}Dy_yCa_xOCl_{1-x}$	Z	2	V (Å³)	115.919	
a=b (Å)	4.1127(5)	*c (Å)*	6.8586(1)	S.G.	*P4/nmm*	
Atom	x	y	z	frac	Wyckoff	Uiso
Dy	0.0029(5)	0.4971(4)	0.1734(9)	0.790	2c	0.0098(8)
La	0.0012(4)	0.5029(5)	0.1756(8)	0.011	2c	0.0043(2)
Ca	0.0009(2)	0.5025(8)	0.1751(7)	0.180	2c	0.0042(3)
O	0.9990(7)	0.0014(3)	0.0013(7)	1.000	2a	0.0132(6)
Cl	0.0011(1)	0.4984(8)	0.6318(9)	0.870	2c	0.0115(7)

Table S2E. Refined lattice parameters, atomic positions and thermal parameters for $La_{0.69}Dy_{0.011}Ca_{0.25}OCl_{0.70}$.

$La_{0.69}Dy_{0.011}Ca_{0.25}OCl_{0.70}$						
wR	10.30%	R	9.06%	χ^2	2.1	
2θ range	6 - 90°	Radiation	Cu Kα 1.5406 Å	Temp	295K	
Formula	$La_{1-x-y}Dy_yCa_xOCl_{1-x}$	Z	2	V (Å³)	115.763	
a=b (Å)	4.1088(9)	*c (Å)*	6.8543(9)	S.G.	*P4/nmm*	
Atom	x	y	z	frac	Wyckoff	Uiso
Dy	0.0030(7)	0.4974(7)	0.1737(4)	0.690	2c	0.0106(8)
La	0.0018(1)	0.5021(3)	0.1755(1)	0.011	2c	0.0051(7)
Ca	0.0014(1)	0.5017(6)	0.1750(8)	0.250	2c	0.0047(6)
O	0.9989(9)	0.0016(9)	0.0015(6)	1.000	2a	0.0140(6)

Cl	0.0012(9)	0.4977(1)	0.6322(7)	0.700	2c	0.0126(8)

Table S2F. Refined lattice parameters, atomic positions and thermal parameters for $La_{0.99}Tb_{0.051}OCl_{1.02}$.

**$La_{0.99}Tb_{0.051}OCl_{1.02}$**						
Wr	10.61%	R	9.30%	χ^2	3.9	
2θ range	6 - 90°	Radiation	Cu Kα 1.5406 Å	Temp	295K	
Formula	$La_{1-x-y}Tb_yCa_xOCl_{1-x}$	Z	2	V (Å³)	116.435	
a=b (Å)	4.1194(5)	*c (Å)*	6.8666(9)	S.G.	*P4/nmm*	
Atom	x	y	z	frac	Wyckoff	Uiso
Tb	0.0025(5)	0.4978(5)	0.1736(3)	0.990	2c	0.0095(6)
La	0.0013(4)	0.5025(2)	0.1752(5)	0.051	2c	0.0052(3)
O	0.9987(9)	0.0012(9)	0.0012(8)	1.000	2a	0.0127(8)
Cl	0.0011(7)	0.4982(4)	0.6324(9)	1.020	2c	0.0118(8)

Table S2G. Refined lattice parameters, atomic positions and thermal parameters for $La_{0.9}Tb_{0.048}Ca_{0.059}OCl_{0.96}$.

**$La_{0.9}Tb_{0.048}Ca_{0.059}OCl_{0.96}$**						
wR	9.81%	R	7.85%	χ^2	2.5	
2θ range	6 - 90°	Radiation	Cu-Kα 1.5406 Å	Temp	295K	
Formula	$La_{1-x-y}Tb_yCa_xOCl_{1-x}$	Z	2	V (Å³)	116.305	
a=b (Å)	4.1164(3)	*c (Å)*	6.8652(4)	S.G.	*P4/nmm*	
Atom	x	y	z	frac	Wyckoff	Uiso
Tb	0.0027(6)	0.4975(3)	0.1733(5)	0.900	2c	0.0099(9)
La	0.0014(5)	0.5024(9)	0.1754(3)	0.048	2c	0.0047(9)
Ca	0.0010(9)	0.5020(7)	0.1749(7)	0.059	2c	0.0044(4)
O	0.9985(7)	0.0018(6)	0.0011(6)	1.000	2a	0.0134(2)
Cl	0.0010(1)	0.4983(3)	0.6319(1)	0.960	2c	0.0122(1)

Table S2H. Refined lattice parameters, atomic positions and thermal parameters for $La_{0.85}Tb_{0.049}Ca_{0.10}OCl_{0.91}$.

**$La_{0.85}Tb_{0.049}Ca_{0.10}OCl_{0.91}$**						
wR	9.81%	R	6.94%	χ^2	4.2	
2θ range	6 - 90°	Radiation	Cu Kα 1.5406 Å	Temp	295K	

Formula	$La_{1-x-y}Tb_yCa_xOCl_{1-x}$	Z	2	V (Å³)	116.008	
a=b (Å)	4.1163(5)	*c (Å)*	6.8591(8)	S.G.	*P4/nmm*	
Atom	x	y	z	frac	Wyckoff	Uiso
Tb	0.0028(2)	0.4972(9)	0.1735(2)	0.850	2c	0.0102(3)
La	0.0016(6)	0.5028(8)	0.1757(1)	0.049	2c	0.0050(1)
Ca	0.0012(8)	0.5023(1)	0.1752(5)	0.100	2c	0.0046(9)
O	0.9986(6)	0.0015(5)	0.0014(3)	1.000	2a	0.0138(7)
Cl	0.0013(8)	0.4978(1)	0.6325(2)	0.910	2c	0.0124(6)

Table S2I. Refined lattice parameters, atomic positions and thermal parameters for $La_{0.75}Tb_{0.052}Ca_{0.19}OCl_{0.87}$.

**$La_{0.75}Tb_{0.052}Ca_{0.19}OCl_{0.87}$**						
wR	9.01%	R	6.23%	χ²	3.8	
2θ range	6 - 90°	Radiation	Cu Kα 1.5406 Å	Temp	295K	
Formula	$La_{1-x-y}Tb_yCa_xOCl_{1-x}$	Z	2	V (Å³)	115.806	
a=b (Å)	4.1133(4)	*c (Å)*	6.8534(4)	S.G.	*P4/nmm*	
Atom	x	y	z	frac	Wyckoff	Uiso
Tb	0.00260(5)	0.4976(9)	0.1734(4)	0.750	2c	0.01070(8)
La	0.00150(7)	0.5022(3)	0.1755(2)	0.052	2c	0.00490(8)
Ca	0.00110(7)	0.5018(1)	0.1750(3)	0.190	2c	0.00450(8)
O	0.9988(6)	0.00170(3)	0.00120(1)	1.000	2a	0.01370(8)
Cl	0.00120(5)	0.4980(6)	0.6327(6)	0.870	2c	0.01250(8)

Table S2J. Refined lattice parameters, atomic positions and thermal parameters for $La_{0.69}Tb_{0.056}Ca_{0.28}OCl_{0.73}$.

**$La_{0.69}Tb_{0.056}Ca_{0.28}OCl_{0.73}$**						
wR	11.02%	R	8.22%	χ²	3.3	
2θ range	6 - 90°	Radiation	Cu Kα 1.5406 Å	Temp	295K	
Formula	$La_{1-x-y}Tb_yCa_xOCl_{1-x}$	Z	2	V (Å³)	115.754	
a=b (Å)	4.1101(1)	*c (Å)*	6.8486(5)	S.G.	*P4/nmm*	
Atom	x	y	z	frac	Wyckoff	Uiso
Tb	0.0029(1)	0.4974(5)	0.1738(1)	0.690	2c	0.0096(8)
La	0.0017(9)	0.5027(6)	0.1759(6)	0.056	2c	0.0053(6)
Ca	0.0013(2)	0.5023(3)	0.1754(9)	0.280	2c	0.0048(1)

O	0.9984(6)	0.0015(8)	0.0013(6)	1.000	2a	0.0131(3)
Cl	0.0011(4)	0.4986(5)	0.6323(5)	0.730	2c	0.0120(9)

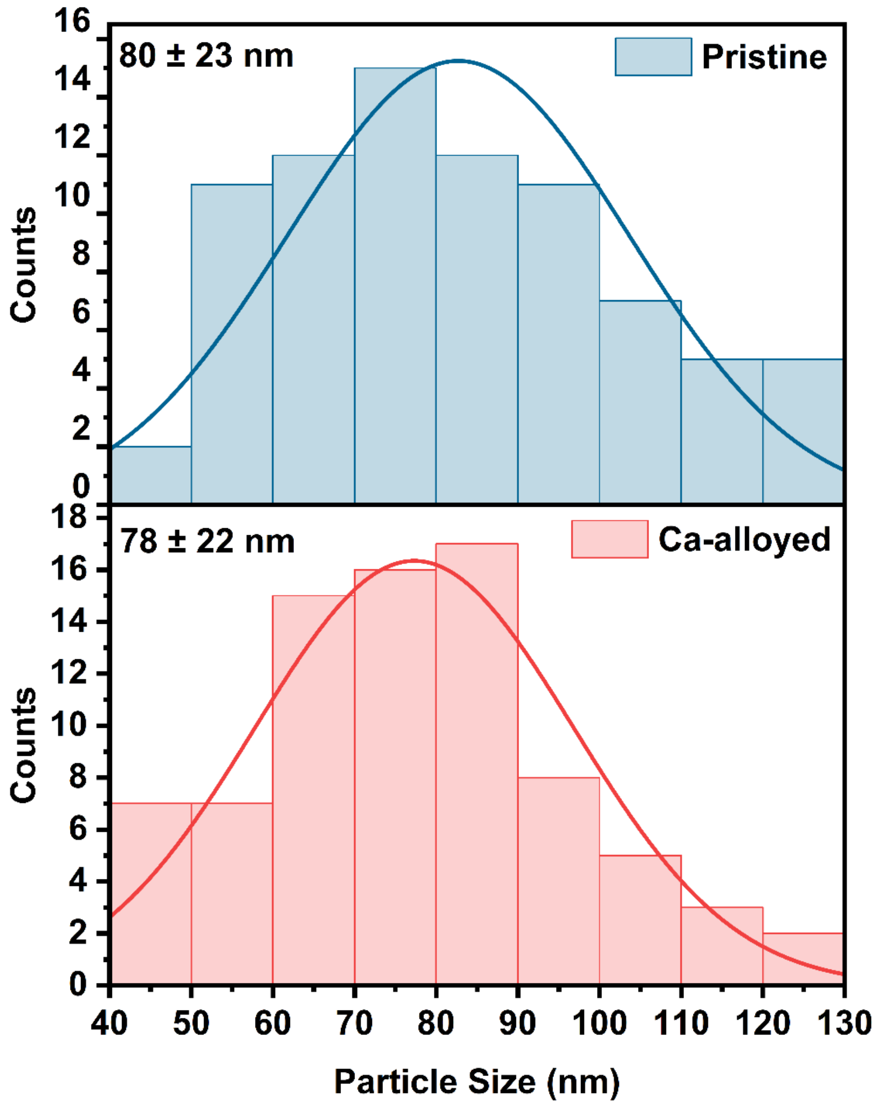

Figure S5. Histograms of particle size distribution of (A) unalloyed and (B) 10 at.% Ca-alloyed LaOCl nanoplatelets determined from TEM images. A total of 160 individual particles were measured to obtain the average size and standard deviation.

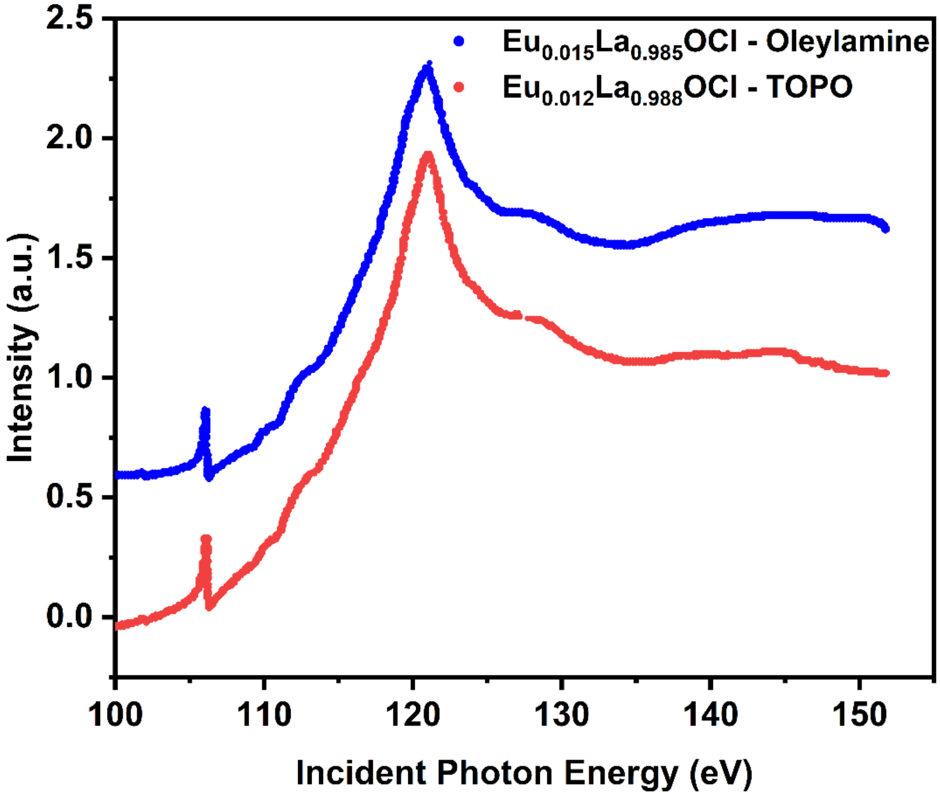

Figure S6. Internal standard of Eu-alloyed LaOCl capped with Oleylamine and TOPO. *Reproduced from (29). Copyright 2018 American Chemical Society.*

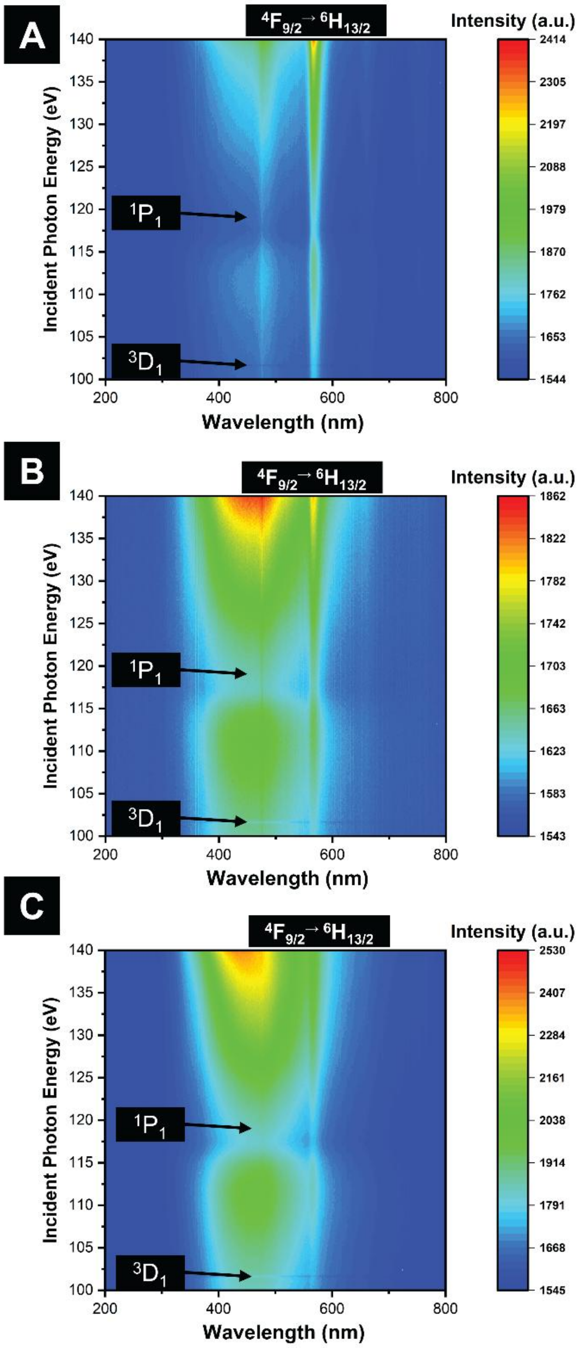

Figure S7. 3D contour map of XEOL intensity as a function of incident photon energy for (A) $La_{0.94}Dy_{0.013}Ca_{0.061}OCl_{0.99}$; (B) $La_{0.89}Dy_{0.013}Ca_{0.10}OCl_{0.94}$; (C) $La_{0.79}Dy_{0.011}Ca_{0.18}OCl_{0.87}$.

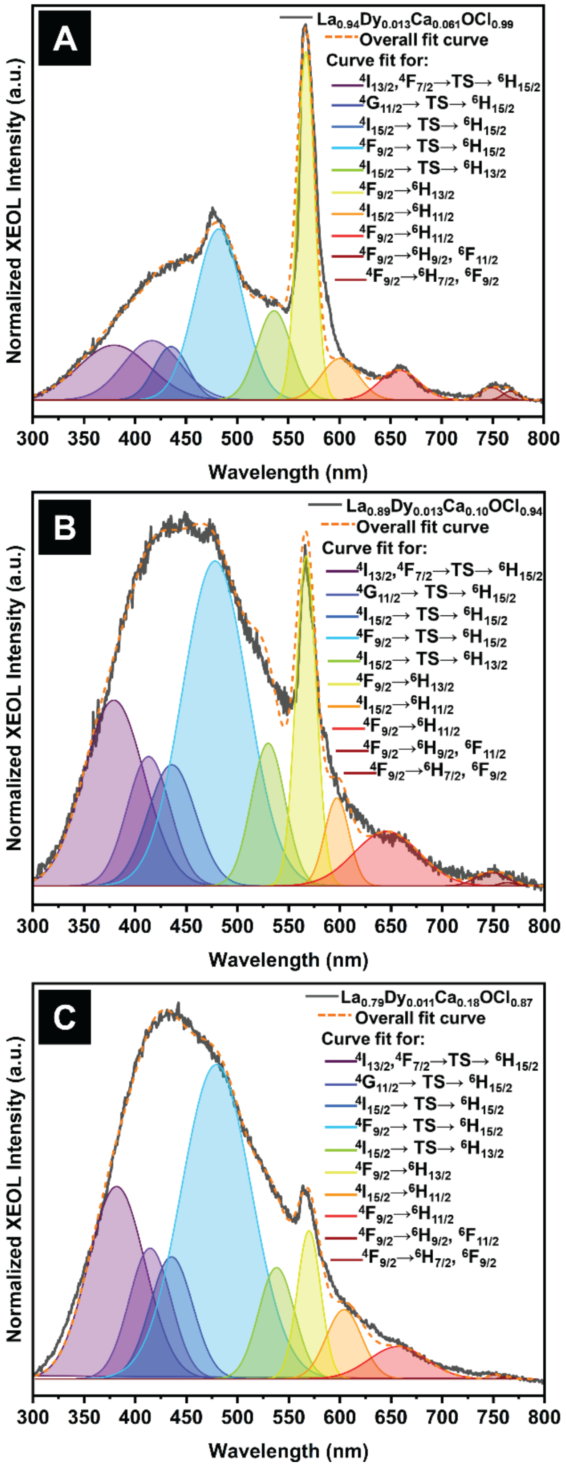

Figure S8. FWHM curve fits of XEOL spectra for (A) $La_{0.94}Dy_{0.013}Ca_{0.061}OCl_{0.99}$; (B) $La_{0.89}Dy_{0.013}Ca_{0.10}OCl_{0.94}$; and (C) $La_{0.79}Dy_{0.011}Ca_{0.18}OCl_{0.87}$ (TS = Trap States).

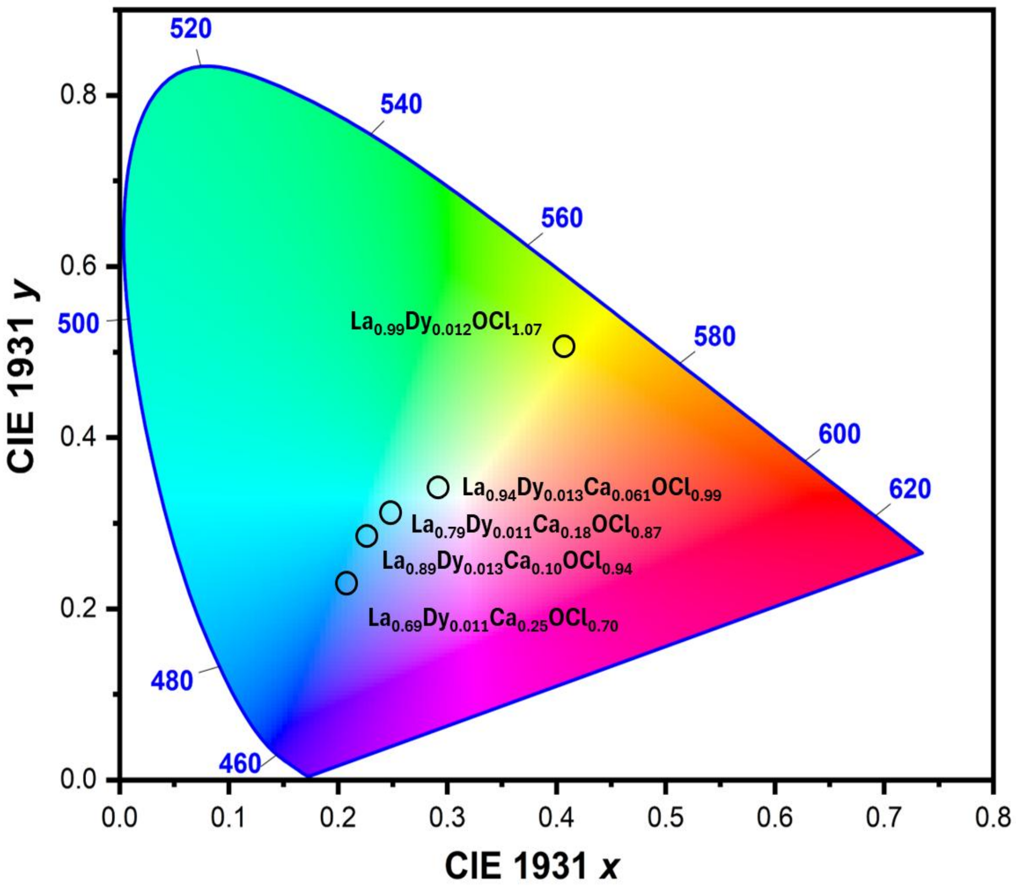

Figure S9. CIE chromaticity diagram indicating the relative positions of the $La_{1-x-y}Ca_xDy_yOCl_{1-x}$.

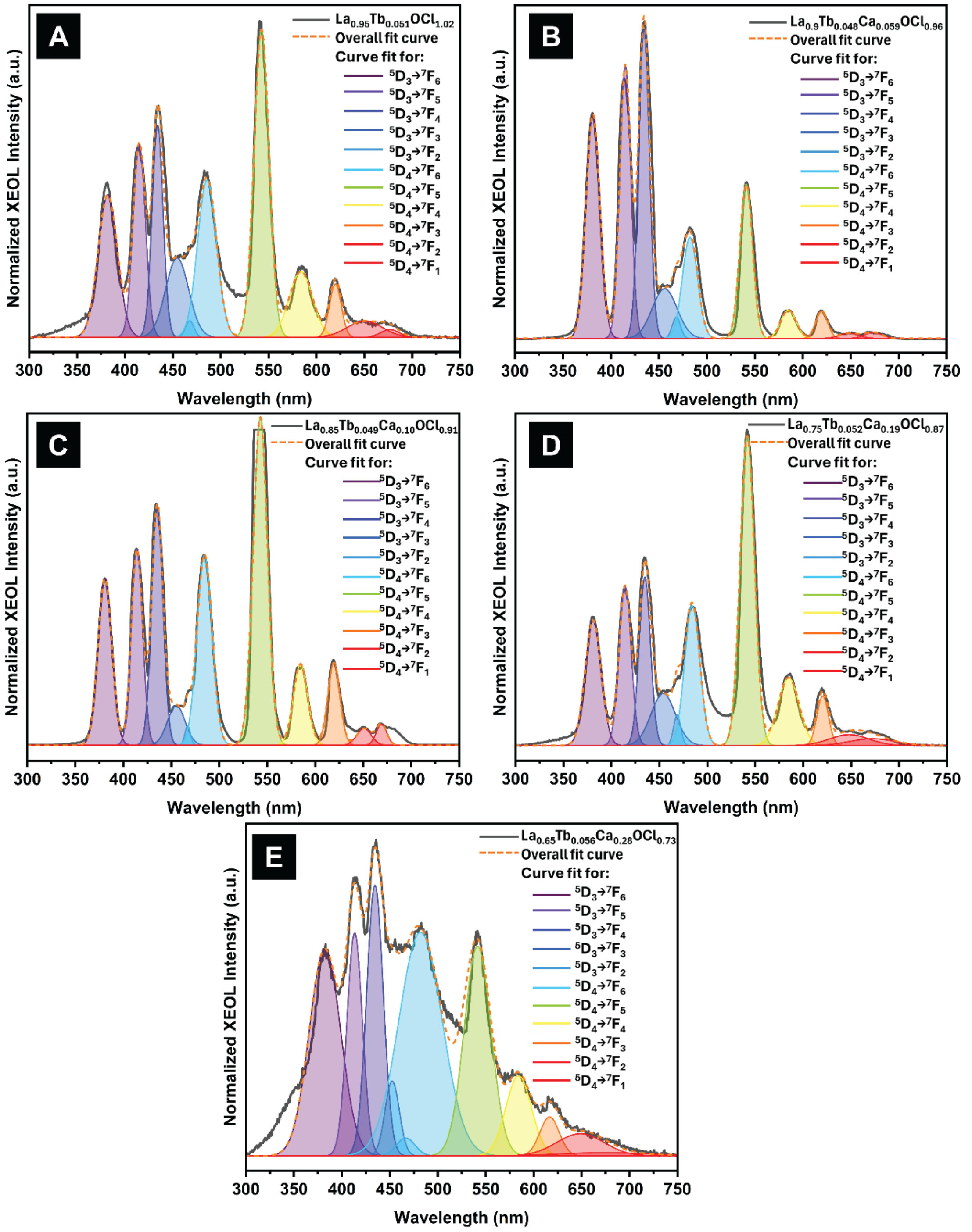

Figure S10. FWHM curve fit of XEOL spectra for (A) $La_{0.99}Tb_{0.051}OCl_{1.02}$; (B) $La_{0.9}Tb_{0.048}Ca_{0.059}OCl_{0.96}$; (C) $La_{0.85}Tb_{0.049}Ca_{0.10}OCl_{0.91}$; (D)$La_{0.75}Tb_{0.052}Ca_{0.19}OCl_{0.87}$; and (E) $La_{0.69}Tb_{0.056}Ca_{0.28}OCl_{0.73}$.

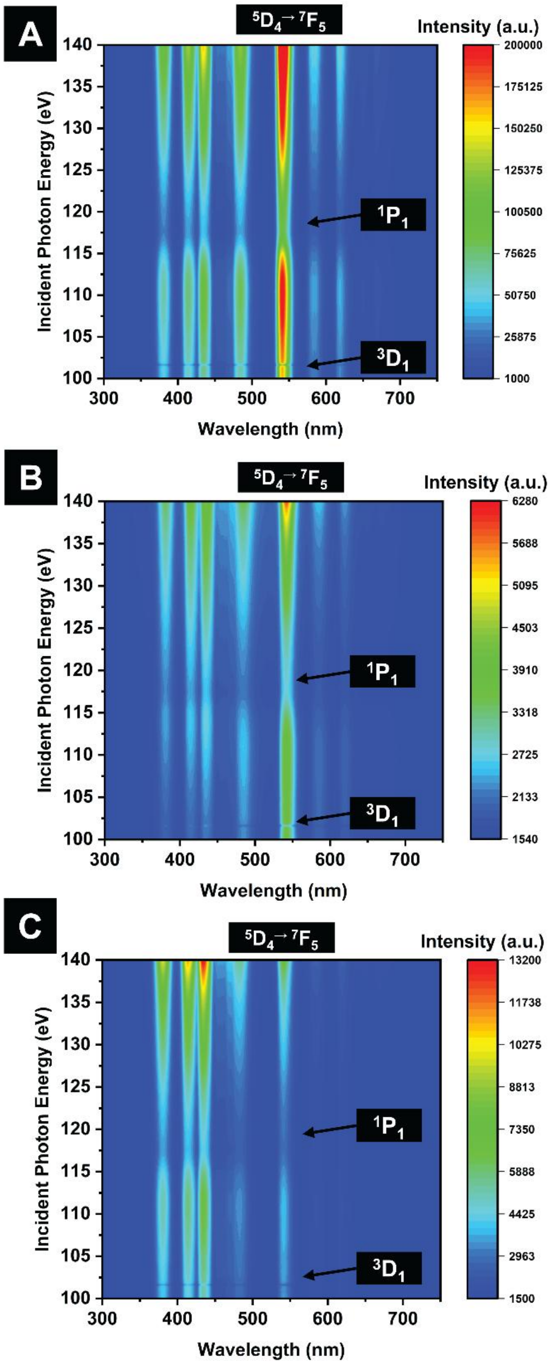

Figure S11. 3D contour map of XEOL intensity as a function of incident photon energy for (A) $La_{0.9}Tb_{0.048}Ca_{0.059}OCl_{0.96}$; (B) $La_{0.85}Tb_{0.049}Ca_{0.10}OCl_{0.91}$; and (C) $La_{0.75}Tb_{0.052}Ca_{0.19}OCl_{0.87}$.

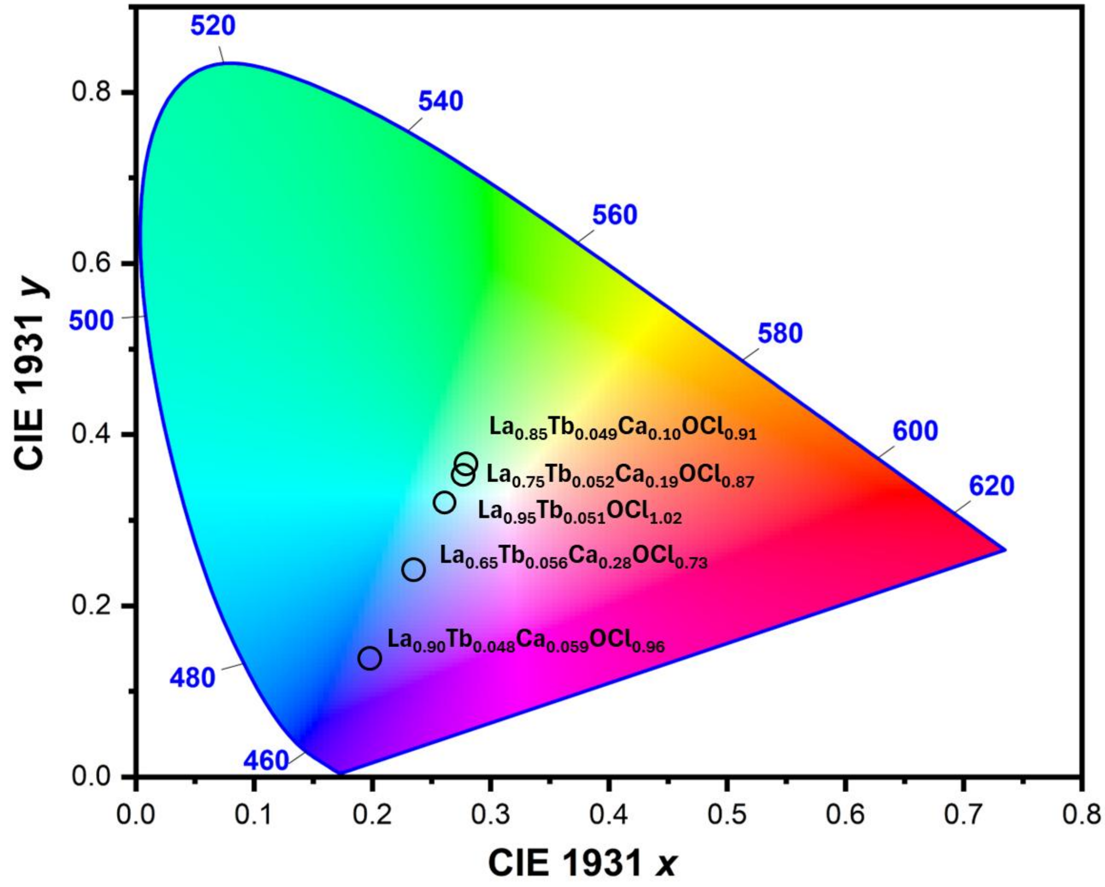

Figure S12. CIE chromaticity diagram indicating the relative positions of the $La_{1-x-y}Ca_xTb_yOCl_{1-x}$.

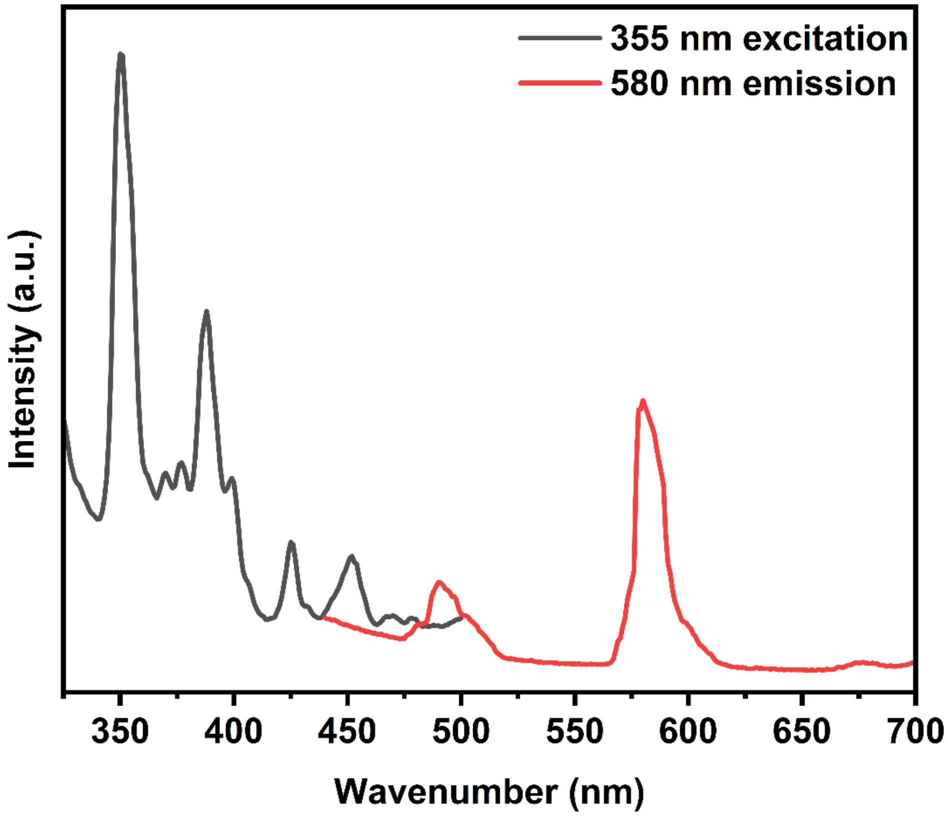

Figure S13. Photoluminescence emission and excitation spectra acquired for $La_{0.99}Dy_{0.012}OCl_{1.07}$ upon excitation at 355 and emission at 580 nm.

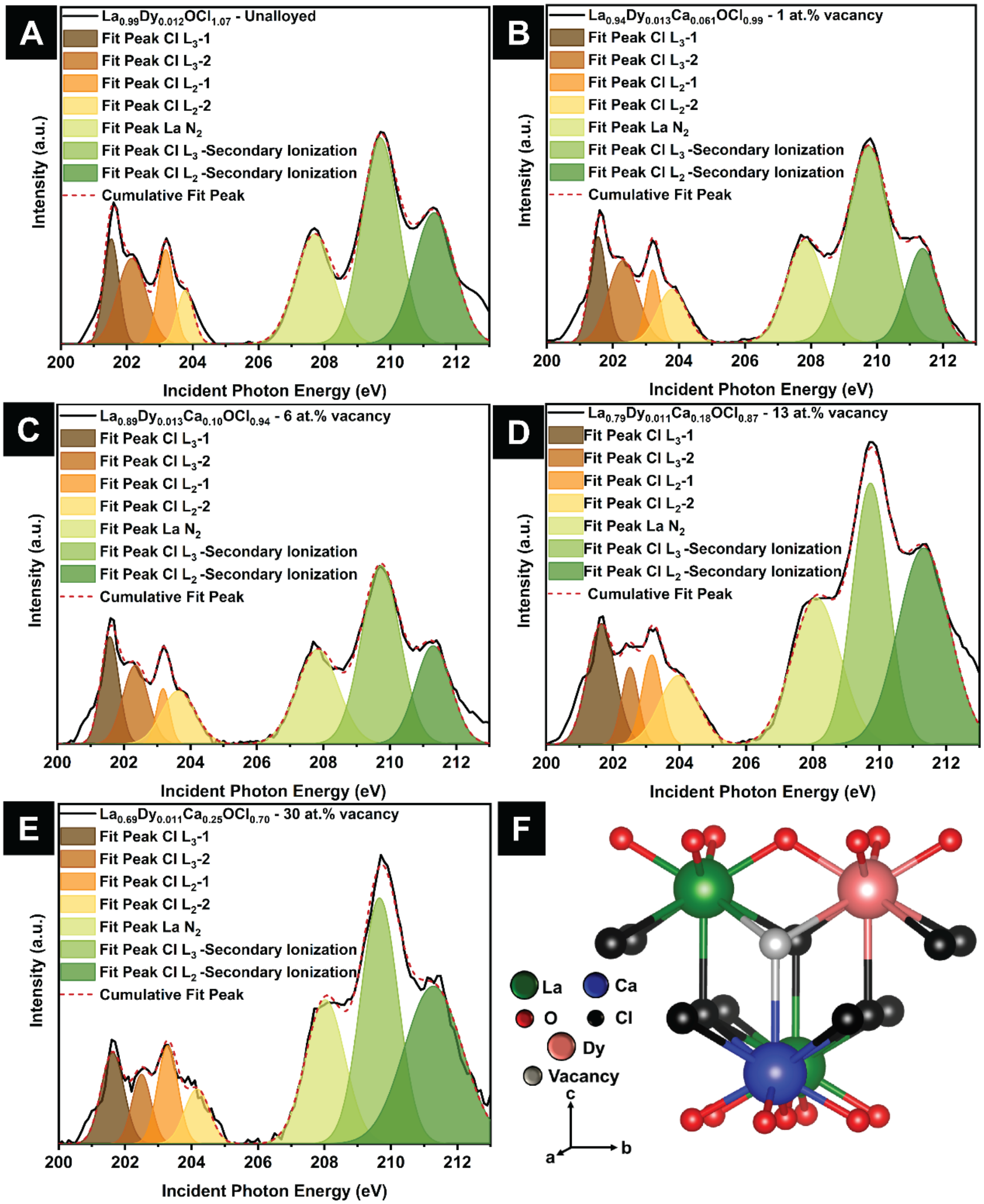

Figure S14. FWHM curve fit of Cl $L_{2,3}$ and La N_2 edge XANES spectra for (A) $La_{0.99}Dy_{0.012}OCl_{1.07}$; (B) $La_{0.94}Dy_{0.013}Ca_{0.061}OCl_{0.99}$; (C) $La_{0.89}Dy_{0.013}Ca_{0.10}OCl_{0.94}$; (D) $La_{0.79}Dy_{0.011}Ca_{0.18}OCl_{0.87}$; and (E) $La_{0.69}Dy_{0.011}Ca_{0.25}OCl_{0.70}$; (F) Structural illustration of Dy - alloyed LaOCl.

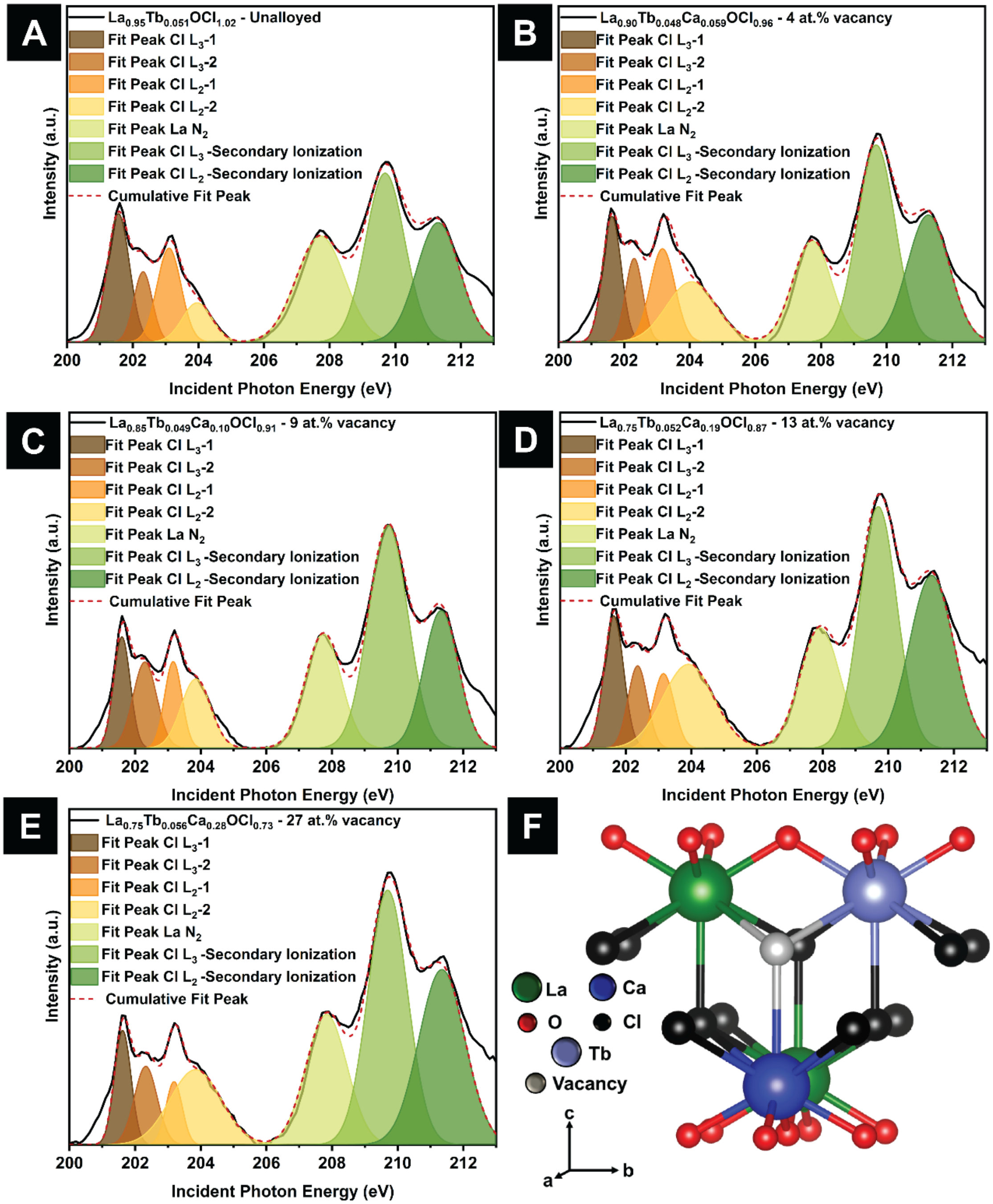

Figure S15. FWHM curve fit of Cl $L_{2,3}$ and La N_2 edge XANES spectra for (A) $La_{0.95}Tb_{0.051}OCl_{1.02}$; (B) $La_{0.9}Tb_{0.048}Ca_{0.059}OCl_{0.96}$; (C) $La_{0.85}Tb_{0.049}Ca_{0.10}OCl_{0.91}$; (D) $La_{0.75}Tb_{0.052}Ca_{0.19}OCl_{0.87}$; and (E) $La_{0.65}Tb_{0.056}Ca_{0.28}OCl_{0.73}$; (F) Structural illustration of Tb-alloyed LaOCl.

Table S3. Analysis of the correlation between FHWM of Cl $L_{2,3}$ edge and defect concentration for $La_{1-x-y}Ca_xDy_yOCl_{1-x}$.

	Defect Concentration (at.%)				
FWHM	0	1	6	13	30
Cl L_3 -1	0.54	0.60	0.81	0.64	0.99
Cl L_3 -2	1.04	1.05	0.68	0.89	0.60
Cl L_2 -1	0.57	0.51	0.72	0.46	0.69
Cl L_2 -2	0.59	1.04	0.89	1.19	1.37
La N_2	1.39	1.38	1.39	1.48	1.71
Cl L_3 – Secondary Ionization	1.32	1.55	1.32	1.37	1.27
Cl L_3 – Secondary Ionization	1.46	1.15	2.07	1.26	1.83
Sum of Cl L_3 edge	1.58	1.65	1.50	1.53	1.58
Sum of Cl L_2 edge	1.16	1.55	1.61	1.66	2.07
Ratio of Cl L_2 and L_3 edge	0.74	0.93	1.08	1.08	1.31

Table S4. Analysis of the correlation between FHWM of Cl $L_{2,3}$ edge and defect concentration for $La_{1-x-y}Ca_xTb_yOCl_{1-x}$.

	Defect Concentration (at.%)				
FWHM	0	4	9	13	27
Cl L_3 -1	0.60	0.80	0.59	0.70	0.67
Cl L_3 -2	0.85	0.66	0.82	0.69	0.62
Cl L_2 -1	0.62	0.86	0.58	0.76	0.89
Cl L_2 -2	1.06	1.07	1.84	1.84	1.78
La N_2	1.29	1.74	1.47	1.44	1.38
Cl L_3 – Secondary Ionization	1.41	1.40	1.41	1.33	1.36
Cl L_3 – Secondary Ionization	1.24	1.55	1.65	1.60	1.51
Sum of Cl L_3 edge	1.44	1.46	1.42	1.40	1.29
Sum of Cl L_2 edge	1.68	1.93	2.42	2.60	2.67
Ratio of Cl L_2 and L_3 edge	1.17	1.32	1.71	1.86	2.08

Table S5. Analysis of the correlation between FHWM of blue and red channels and defect concentration for $La_{1-x-y}Ca_xDy_yOCl_{1-x}$.

	Defect Concentration (at.%)				
FWHM	0	1	6	13	30
$^4I_{13/2}$,$^4F_{7/2}$→TS→$^6H_{15/2}$	18.97	69.47	65.06	82.45	65.19
$^4G_{11/2}$→ TS→$^6H_{15/2}$	12.93	49.47	48.26	66.25	56.49
$^4I_{15/2}$→ TS→$^6H_{15/2}$	16.20	54.77	48.99	39.27	53.37
$^4F_{9/2}$→ TS→$^6H_{15/2}$	25.64	74.58	80.24	54.43	87.28
$^4I_{15/2}$→ TS→$^6H_{13/2}$	11.77	38.85	41.21	39.75	45.44
$^4F_{9/2}$→$^6H_{13/2}$	17.55	23.55	27.08	20.07	31.99
$^4I_{15/2}$→$^6H_{11/2}$	21.90	28.16	40.98	42.30	60.31
$^4F_{9/2}$→$^6H_{11/2}$	23.02	75.52	66.29	46.22	33.53
$^4F_{9/2}$→$^6H_{9/2}$, $^4F_{11/2}$	11.96	35.32	11.77	24.92	11.77
$^4F_{9/2}$→$^6H_{7/2}$, $^4F_{9/2}$	12.47	17.66	11.77	17.90	11.77
Sum of blue channels	85.50	287.13	283.76	282.15	307.77
Sum of red channels	86.90	180.21	157.90	151.40	149.38
Ratio of blue and red channels	0.98	1.59	1.80	1.86	2.06

Table S6. Analysis of the correlation between FHWM of blue and red channels and defect concentration for $La_{1-x-y}Ca_xTb_yOCl_{1-x}$.

	Defect Concentration (at.%)				
FWHM	0	4	9	13	27
$^5D_3 \rightarrow ^7F_6$	23.92	17.05	16.99	19.60	38.50
$^5D_3 \rightarrow ^7F_5$	15.10	13.45	13.71	14.75	19.04
$^5D_3 \rightarrow ^7F_4$	13.41	13.19	13.83	13.85	25.45
$^5D_3 \rightarrow ^7F_3$	28.45	30.48	21.49	29.97	19.61
$^5D_3 \rightarrow ^7F_2$	10.35	10.06	6.76	8.67	25.82
$^5D_4 \rightarrow ^7F_6$	23.26	18.56	18.44	18.54	43.27
$^5D_4 \rightarrow ^7F_5$	18.37	15.53	17.63	17.50	32.97
$^5D_4 \rightarrow ^7F_4$	28.65	18.75	17.57	25.82	21.42
$^5D_4 \rightarrow ^7F_3$	14.33	14.48	14.88	14.03	23.77
$^5D_4 \rightarrow ^7F_2$	33.36	24.00	17.66	18.83	18.48
$^5D_4 \rightarrow ^7F_1$	22.80	21.26	6.83	5.46	5.88
Sum of blue channels	91.24	84.23	72.77	86.83	128.42
Sum of red channels	140.77	112.58	93.01	100.17	145.79
Ratio of blue and red channels	0.65	0.75	0.78	0.87	0.88
